# Unveiling Chemical-Microbial Cascade Risk Factors from Plastic Pipe Leaching in Drinking Water

**DOI:** 10.1021/acs.est.5c10244

**Published:** 2025-10-14

**Authors:** Mengqing Fan, Ziqian Wang, Mingchen Yao, Xiaoming Li, Walter van der Meer, Yu Tao, Joan B. Rose, Gang Liu

**Affiliations:** † Key Lab of Aquatic Chemistry, State Key Lab of Regional Environment and Sustainability, 26442 Research Centre for Eco-Environmental Sciences, Chinese Academy of Sciences, Beijing 100085, China; ‡ University of Chinese Academy of Sciences, Beijing 100049, China; § Science and Technology, University of Twente, P.O. Box 217, Enschede 7500AE, The Netherlands; ∥ School of Eco-Environment, 529484 Harbin Institute of Technology, Shenzhen 518055, China; ⊥ Department of Fisheries and Wildlife, Michigan State University, East Lansing, Michigan 48823, United States; # Sanitary Engineering, Department of Water Management, Faculty of Civil Engineering and Geosciences, 2860 Delft University of Technology, P.O. Box 5048, GA Delft 2600, The Netherlands

**Keywords:** plastic leaching, FT-ICR MS, dissolved organic matter molecules, metagenomics, microbial risks

## Abstract

Plastic pipes are increasingly used in drinking water distribution systems, yet their impact on water quality remains insufficiently understood. Here, we systematically investigate the dual outcomes posed by plastic pipes—chemical leaching and cascaded microbial exposure risks—by integrating Fourier Transform Ion Cyclotron Resonance Mass Spectrometry and metagenomic analysis. Our results reveal that plastic pipes continuously release dissolved organic matter (DOM), including organic additives such as bisphenols (BPs) and organophosphate esters (OPEs), which profoundly reshape microbial communities. Under chlorinated conditions, leached DOM alters microbial diversity, promoting chlorine-resistant bacteria and opportunistic pathogens (OPs), while under nonchlorinated conditions, it accelerates microbial growth and enriches antibiotic resistance genes (ARGs), OPs, and virulence factors (VFs). Among plastic materials, polyethylene (PE) exhibited the highest chemical risk, releasing high concentrations of TCPP (700 ng/L) and BPF (200 ng/L) along with 207–227 unique DOM molecules. In contrast, polyvinyl chloride (PVC) supported the highest OP abundance, while polypropylene random copolymer (PPR) fostered the greatest OP diversity. These findings challenge conventional drinking water safety assessments that separate chemical contamination from microbial risk, underscoring the urgent need for an integrated risk assessment framework. Furthermore, they highlight the necessity of paying greater attention to the chemical and cascading microbial issues arising from the leaching of plastic pipes into drinking water, and of conducting a more comprehensive assessment of the associated potential health risks.

## Graphical abstract

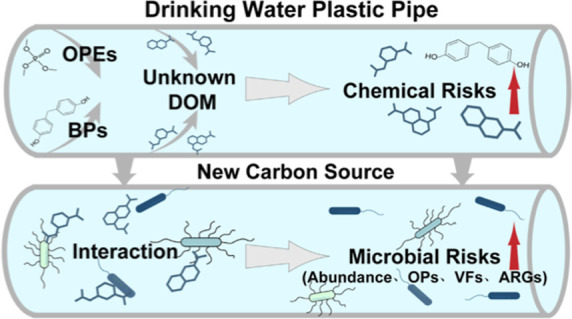

## 1 Introduction

Plastic pipes are increasingly replacing traditional materials in drinking water distribution systems (DWDSs) worldwide due to their lightweight properties, corrosion resistance, and low maintenance costs. Compared to metal pipes (e.g., copper, cast iron), plastic alternatives such as polyvinyl chloride (PVC), polypropylene random copolymer (PPR), and polyethylene (PE) effectively mitigate heavy metal leaching and scaling.^1^ Life cycle assessments indicate that PVC pipes used in premise plumbing consume 86% and 91% less energy than galvanized steel and copper pipes, respectively, based on energy use across production and transportation stages.^2^ In the United States, the EPA’s 2023 Drinking Water Infrastructure Needs Survey and Assessment estimates that one-third of existing pipes are plastic, with 54% of planned replacements set to use plastic materials,^3^ with PVC being the most commonly used plastic pipe in drinking water systems.^4^ Similarly, in The Netherlands, over 54.5% of water mains (120,000 km pipelines) are now composed of PVC,^5^ with increasing adoption of PVC and PE in new installations and renovations.^6,7^ As plastic pipes continue to play an expanding role in DWDSs, it is crucial to assess their impact on water quality and safety.

Despite their advantages, plastic materials can release chemical substances into water during use.^8,9^ They are composed of the essential polymer mixed with a complex blend of substances, and the list of plastic-associated chemicals has grown to over 16,000 compounds.^10^ Additives such as bisphenols (BPs) and organophosphate esters (OPEs) are particularly prone to leaching due to their weak binding to the polymer matrix.^11^ BPs are known endocrine disruptors,^12–14^ while OPEs have been linked to neurotoxicity and reproductive toxicity.^15,16^ Beyond additives, plastics also leach DOM, including oligomers and monomers, which can alter water chemistry^17,18^ and introduce new risks.^19,20^ For example, monomers from polystyrene have been shown to induce oxidative stress and cytotoxic effects.^21,22^ Advanced analytical techniques such as excitation–emission matrix fluorescence spectroscopy combined with parallel factor analysis (EEM-PARAFAC) have proven effective in characterizing plastic-leached DOM,^23–25^ while Fourier transform ion cyclotron resonance mass spectrometry (FT-ICR MS) provides high-resolution insights into its molecular composition.^26,27^ However, significant knowledge gaps persist regarding the outcomes of plastic leaching in drinking water, including the temporal dynamics of plastic-leached additives (e.g., BPs and OPEs) and the molecular composition of DOM.

The continuous release of DOM from plastic pipes introduces a new organic carbon source into drinking water, potentially stimulating microbial activity and reshaping microbial community composition—a phenomenon widely documented in marine and freshwater ecosystems.^28–30^ In DWDSs, fluctuating hydraulic retention times create conditions for dynamic interactions between plastic-leached DOM and microbial communities. Studies indicate that pipe material influences microbial composition in both bulk water and biofilms,^31^ while factors such as chlorination and heating can accelerate the release of microplastics and DOM from PPR pipes, increasing microbial toxicity risks.^32^ However, how chemical leaching from plastic pipes contributes to microbial risks remains poorly understood, presenting a fundamental challenge in evaluating their overall impact on drinking water safety.

Residual disinfectants such as chlorine in DWDSs introduce a complex interplay between plastic pipe leaching and microbial risks. Chlorine’s strong oxidative properties may accelerate plastic aging, altering the composition of leached DOM and potentially forming more toxic byproducts, such as halobenzoquinones and haloacetic acids.^33,34^ Conversely, chlorine may mitigate some microbial risks by suppressing microbial growth and diversity. To systematically assess the chemical-microbial risks associated with plastic pipes in drinking water, this study employed a laboratory-based static simulation experiment. Over a 30 day period, we monitored the dynamic changes in key plastic additives (BPs, OPEs) and DOM spectral characteristics leached from PVC, PPR, and PE under chlorinated and nonchlorinated conditions. By integrating FT-ICR MS and metagenomic analysis, we characterized the molecular composition of plastic-leached DOM and its regulatory effects on microbial community dynamics, antibiotic resistance genes (ARGs), opportunistic pathogens (OPs), and virulence factors (VFs). This study provides an initial understanding of the intertwined chemical and microbial risk factors posed by plastic pipes in DWDSs, offering critical insights for risk assessment and management.

## 2 Materials and Methods

### 2.1 Plastic Materials

This study focused on three commonly used plastic materials: PVC, PPR and PE. These materials were manufactured in accordance with relevant Chinese standards for water supply pipes to ensure compliance and drinking water approval. The standards include GB/T 10002.1–2006 for PVC, GB/T 18742.2–2017 for PPR and GB/T 13663.2–2018 for PE. The pipes used in this study were sourced from the local market and thoroughly rinsed three times with Milli-Q water prior to use. The pipes had a consistent length of 1.5 m and outer diameter of 75 mm, while their wall thickness varied according to the respective standards: 3.6 mm for PVC, 4.5 mm for PE and 6.9 mm for PPR. Custom-made polytetrafluoroethylene (PTFE) plugs with sampling ports (accessible for opening) were installed at both ends of the pipes to prevent external contamination and facilitate sample collection. Initial tap water was collected from the laboratory of the Research Center for Eco-Environmental Sciences, Chinese Academy of Sciences, Beijing, China. To ensure that fresh tap water was obtained from the network, the taps were opened and allowed to run for 15 min prior to sampling until a constant temperature was reached. Water quality information is in Table S2.

### 2.2 Experimental Setup and Sampling

The experiment was divided into two distinct treatments to simulate the migration of substances from plumbing plastics into water under both chlorinated and chlorine-free conditions. These two treatments were conducted simultaneously using separate sets of pipes to avoid any cross-contamination. A simplified schematic of the setup is provided in Supporting Information Figure S1. In the first treatment containing Cl, fresh tap water was added, with residual chlorine levels monitored every 2 days and maintained between 0.3 and 1 mg/L by adding NaClO solution. This range was determined based on previous large-scale survey on Beijing water treatment plants and also falls within the current national standards for residual chlorine concentration in drinking water in China (0.3–2 mg/L). In the second treatment without Cl, tap water was pretreated with ascorbic acid to quench chlorine before being introduced into the pipes, maintaining chlorine-free conditions during the experiment. After filling the pipes with water, both ends were sealed with customized PTFE plugs and maintained at 25 ± 5 °C for 30 days. Since PTFE possesses extremely high chemical stability,^35^ it is commonly regarded in research as a material that does not introduce background interference.^36,37^ Meanwhile, chlorinated and chlorine-free water were added to 5 L glass bottles to serve as controls. Each condition was tested in triplicate (*n* = 2 chlorine conditions × (3 material types + 1 control) × 3 replicates). Sampling was performed over a 30 day period with 5 day intervals on days 0, 5, 10, 15, 20, 25, and 30, respectively. On day 0, fresh tap water samples were collected immediately, followed by regular sampling on the following sampling days. Water samples were extracted using precleaned glass bottles and analyzed on the same day for parameters such as total cell counts (TCC), total organic carbon (TOC), and excitation–emission matrices (EEM), with organic additives extracted and concentrated. The samples were collected in glass bottles, filtered through GF/F membranes (0.22 μm), and stored at 4 °C for a maximum of 24 h prior to processing. Based on the regular sampling results and for optimized measurement accuracy, samples for FT-ICR MS and metagenome analysis were collected exclusively at the beginning (day 0) and end of the experiment (day 30).

All glassware used in the experiments were acid-washed overnight, extensively rinsed with Milli-Q water, and heated sterile at 120 °C or combusted at 450 °C for 6 h. All materials associated with microbiological indicators were sterilized.

### 2.3 Characterization of Plastic Organic Additives from the Plastic Pipes

Nine organophosphate esters (OPEs) were analyzed, including tripropyl phosphate, TPP; triisobutyl phosphate, TiBP; tri-*n*-butyl phosphate, TnBP; tris (2-chloroethyl) phosphate, TCEP; tris (1-chloro-2-propyl) phosphate, TCPP; tris (1,3-dichloro-2-propyl) phosphate, TDCP; triphenyl phosphate, TPhP; 2-ethylhexyl diphenyl phosphate, EHDPP; tris­(2-ethylhexyl) phosphate, TEHP) and 7 BPs (bisphenol A, BPA; bisphenol AF, BPAF; bisphenol AP, BPAP; bisphenol F, BPF; bisphenol P, BPP; bisphenol S, BPS; bisphenol Z, BPZ). The CAS numbers, monoisotopic masses, and log *K*_ow_ values for all compounds are presented in Table S3.

#### 2.3.1 Sample Extractions

300 mL water samples were spiked with 100 ng of BPA-D16 and 50 ng each of TnBP-d27 and TPHP-d15 as internal standards. HLB cartridges (500 mg, 6 mL, Waters) were preactivated with 6 mL of methanol followed by 6 mL of ultrapure water. Samples were then passed through the cartridges at a flow rate of 5 mL/min. To remove salts, the cartridges were rinsed with 10 mL of ultrapure water and subsequently dried with nitrogen gas. Elution was carried out using 6 mL of methanol/acetonitrile (1:1) at a flow rate of 1 mL/min. The eluted samples were evaporated to dryness under nitrogen, redissolved in 0.4 mL of methanol, and stored in the dark at −20 °C. Subsequently, 0.6 mL of ultrapure water was added, and the mixture was shaken thoroughly before testing.

#### 2.3.2 Instrumental Analysis

Plastic organic additives were quantified using a Shimadzu high-performance liquid chromatograph (HPLC) coupled with an AB Sciex QTRAP 6500+ mass spectrometer (MS) equipped with a TurboIonSpray electrospray ionization (ESI) source. Positive electrospray ionization (ESI+) was applied for OPEs analysis, while negative electrospray ionization (ESI^–^) was used for BPs analysis. The detailed liquid chromatography conditions and the multiple reaction monitoring (MRM) ion pairs for the target analytes and are provided in Text S1 and Table S4.

#### 2.3.3 Quality Assurance/Quality Control

To reduce background contamination, plasticware like syringes and SPE pipes was prewashed with hexane and methanol, while glass bottles and tubes were baked at 450 °C for 6 h. Procedural blanks were analyzed every 10 samples to check for contamination. No blank corrections were made because blank concentrations were always lower than 10% of the average target additives concentrations in samples. Recovery tests involved spiking water with 9 OPEs and 7 BPs at 100 ng, performed in triplicate. The recoveries ranged from 78.58% to 137.86% for OPEs and from 71.5% to 139.16% for BPs. The method detection limits (MDLs) were calculated based on a signal-to-noise ratio of 3 and further adjusted according to the sample extraction volume. Details on recoveries and MDLs are provided in Table S4.

### 2.4 Dissolved Organic Matter Quantification and Fluorescence Analysis

Water samples were prefiltered using a 0.22 μm GF/F filter membrane. DOM concentrations were measured using a Shimadzu TOC-L analyzer, with a detection limit of 0.01 mg L^–1^ and ±2% precision. UV absorbance at 254 nm was determined using a Cary 60 UV–visible spectrophotometer (Agilent, US) with a 1 cm quartz cell. As the absorbance of all samples at 254 nm was below 0.3,^38^ the fluorescence excitation–emission matrices (EEMs) were directly measured using the F-7000 fluorescence spectrometer (Hitachi, Japan). Excitation wavelengths (Ex) ranged from 200 to 450 nm at 5 nm intervals, and emission (Em) wavelengths ranged from 220 to 600 nm at 2 nm intervals. Photomultiplier detector voltage and scan speed were fixed at 700 V and 12,000 nm/min, respectively. The EEM data were analyzed using the StaRDOM package in R,^39^ including blank subtraction, Raman normalization, the removal of Rayleigh and Raman scattering, etc. Additionally, parallel factor analysis (PARAFAC) was conducted to isolate four fluorescence components (C1–C4) in the samples, which were subsequently uploaded to the OpenFluor online spectral library (https://openfluor.lablicate.com) to identify quantitatively similar spectra. The minimum excitation and emission similarity scores both exceed 0.95.

### 2.5 FT-ICR MS Analysis

#### 2.5.1 Sample Preparation and Measurement

The collected samples were acidified with formic acid (HCOOH, to pH 2.0) and subjected to solid-phase extraction (SPE). The Agilent Bond Elut-PPL cartridges (500 mg per 6 mL) were preactivated with 12 mL of methanol, followed by 12 mL of acidified ultrapure water (HCOOH, pH 2). The samples were then passed through the PPL cartridges at a flow rate of 5 mL/min. After loading, the cartridges were rinsed with 12 mL of acidified ultrapure water to remove salts and dried under a stream of nitrogen gas. The retained compounds were eluted with 12 mL of methanol, and the eluates were blow-dried with nitrogen, redissolved in 1 mL of methanol, and stored in the dark at −20 °C. Notably, unlike previous studies, formic acid (HCOOH) was used instead of hydrochloric acid (HCl) during the SPE process to prevent the formation of chloride adducts between chloride ions and DOM.^40,41^ The molecular composition of DOM in water samples was measured with high accuracy using a 15.0 T Bruker Solarix FT-ICR MS system (Bruker Daltonics, Billerica, MA), equipped with a negative electrospray ionization (ESI) ion source. Details on analytical conditions are provided in Text S2.

#### 2.5.2 Data Analysis

Internal recalibration is an essential step for obtaining FT-ICR MS spectra with ultrahigh mass accuracy (<1.0 ppm). All FT-ICR MS spectra were recalibrated internally using the FTMSCalibrate algorithm.^42^ Peaks with a signal-to-noise ratio (S/N) ≥ 4 were identified, and molecular formulas were assigned using the FTMSDeu algorithm.^43^ Details on molecular formula assignments and molecular parameters are provided in Text S3. Compounds criteria of molecular for molecular formula categorization in Table S5.

### 2.6 Bacterial Quantification and Metagenomic Analysis

#### 2.6.1 Total Cell Count (TCC)

Total bacterial cells in water samples were rapidly enumerated using a flow cytometer, following established protocols.^44–46^ Briefly, 1 mL of sample was stained with 10 μL mL^–1^ SYBR Green I (1:100 dilution in DMSO) and incubated for 10 min at 35 °C in the dark before measurement. Flow cytometric analysis was performed using an Agilent NovoCyte 1040 (NovoCyte, USA).

#### 2.6.2 DNA Extraction, Sequencing and Quality Control

Water samples were filtered through 0.22 μm membranes (Whatman, UK) and stored at −80 °C until DNA extraction using the FastDNA SPIN Kit for Soil (MP Biomedicals, USA) following the manufacturer’s instructions. Metagenomic sequencing was performed on the Illumina HiSeq NovaSeq 6000 platform (Guangdong Magigene Biotechnology Co., Ltd.), with data deposited in the NCBI database under accession code PRJNA1230761. Raw reads were processed using fastp v0.20.0 ^47^ to remove adapters and low-quality sequences (length < 50 bp, quality score < 20, or containing N bases).

#### 2.6.3 Metagenomic Assembly and Bioinformatics

Quality-filtered reads were assembled with MEGAHIT v1.1.2,^48^ and contigs ≥300 bp were retained for downstream analysis. Open reading frames (ORFs) were predicted from the assembled contigs using Prodigal (v2.6.3), and sequences ≥100 bp were retained for translation into amino acids. CD-HIT (v4.6.1) was used to remove redundant sequences with a 90% identity and coverage threshold to generate a nonredundant gene set. High-quality reads were mapped to this gene set with a 95% identity cutoff, and relative abundance (TPM, Transcripts Per Million) was determined by CoverM (v0.6.1).^49^ Taxonomic annotation was performed by aligning representative genes against the NCBI nr database using diamond (v2.1.8).^50^ Human opportunistic pathogens (OPs) were identified based on the 538 human pathogenic species list,^51,52^ as detailed in Table S7. Virulence factors (VFs) and antibiotic resistance genes (ARGs) were obtained by aligning against the VFDB and CARD databases respectively using Diamond, with an *e*-value cutoff ≤ 1 × 10^–5^.

### 2.7 Statistical Analysis

We categorized the organic additives into four ranks based on temporal variations to comprehensively understand their concentration changes: Rank 1 indicates concentrations that never decreased over the study period; rank 2 indicates concentrations that fluctuated but were higher on day 30 than on day 0; rank 3 indicates concentrations that fluctuated but were lower on day 30 than on day 0; rank 4 indicates compounds that were not detected (note: considering the inevitable errors in the solid-phase extraction process, a concentration change of more than 20% is required to be defined as an increase or decrease).

For fluorescent components, to investigate the effect of pipe material on DOM leaching, we compared each plastic pipe group with the control under both chlorinated and nonchlorinated conditions using a two-tailed Wilcoxon rank-sum test to analyze differences in fluorescent components. The effect of chlorine was assessed by comparing the concentrations of fluorescent components between chlorinated and nonchlorinated treatments within the same pipe material group.

To study the DOM molecules introduced by plastics, we excluded molecules that were present in the initial water samples and subtracted blanks. We then characterized the plastic-leached DOM by analyzing the elemental composition, chemical categories, and molecular traits of the introduced molecules.

Co-occurrence network analysis was conducted to explore the relationship between plastic-enriched pathogens and DOM molecules. Spearman correlations were calculated using pathogen relative abundance and molecular relative peak intensity. We first selected plastic-enriched pathogens with average relative abundances greater than 0.01%. These pathogens were required to show enrichment in at least two plastic pipes under both chlorinated and nonchlorinated conditions. Rare molecules (relative abundance < 0.01%) were excluded, and only DOM molecules present in over half of the samples were considered. *P*-Values were adjusted using the Benjamini-Hochberg method to control false discovery rates.^53^ Networks were constructed based on strong correlations (|ρ| ≥ 0.7, *P* < 0.05) and visualized in Gephi (https://gephi.org). All the statistical analyses were performed in R (version 4.3.1, R Core Team, 2023).

## 3 Results

### 3.1 Targeted Additives Leached from Plastic Pipes into Drinking Water

As shown in Figure 1a, the concentrations of 13 out of 16 monitored organic additives from the plastic pipes increased over time in water, including five bisphenols (BPs: BPS, BPF, BPA, BPAF, BPP) and eight organophosphate esters (OPEs: TCEP, TCPP, TiBP, TDCP, TnBP, TPhP, EHDPP, TEHP). Regardless of pipe material or chlorine presence, the cumulative release concentrations of these additives remained below parts per million (ppm) levels.

**Figure 1:**
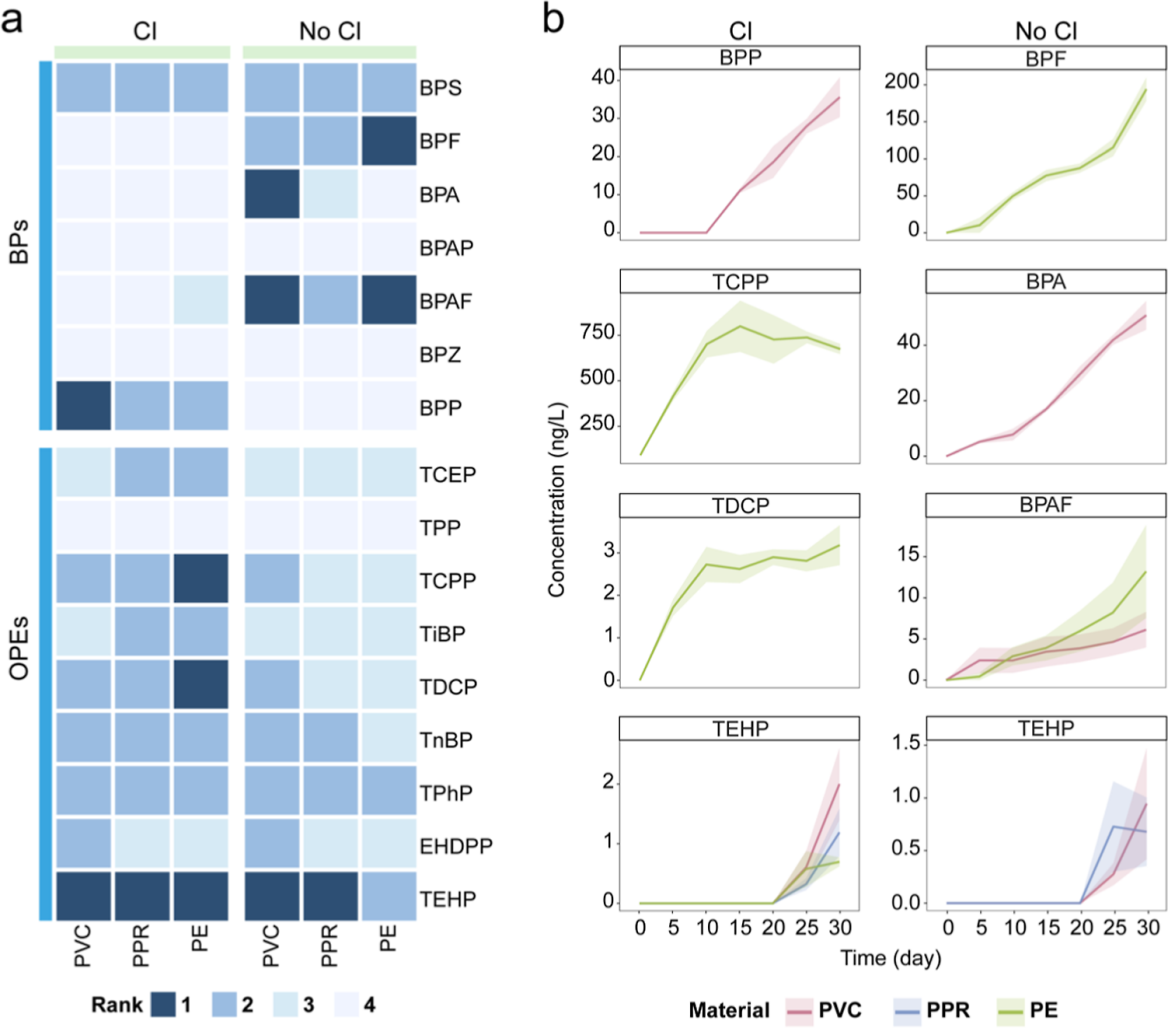
Temporal trends of targeted organic additives from the plastic pipes in water after 30 days exposure to different pipe materials with and without chlorination. (a) Classification of concentration trends: rank 1 indicates concentrations that never decreased over the study period; rank 2 indicates concentrations that fluctuated but were higher on day 30 than on day 0; rank 3 indicates concentrations that fluctuated but were lower on day 30 than on day 0; rank 4 indicates compounds that were not detected. (b) Temporal concentration changes of rank 1 additives, with and without chlorination. Lines with shaded areas in (b) represent means ± standard error, based on triplicate samples (*n* = 3). Note that the shaded areas between points merely connect the standard errors of the measured points for visual guidance and do not involve any calculation or extrapolation.

Most organic additives were detected across all pipe materials, except for BPA, which leached from PVC and PPR but was undetected in PE. Chlorination generally reduced the concentration of most BPs. Notably, BPF, BPA, and BPAF exhibited cumulative increases in nonchlorinated water (BPF in PE: 194 ng/L; BPA in PVC: 50 ng/L; BPAF in PE: 13 ng/L; BPAF in PVC: 6 ng/L), but were not detected in the chlorinated group, suggesting that they may have reacted with chlorine to form transformation products. In contrast, nonchlorinated conditions led to lower concentrations of most OPEs, possibly due to microbial degradation. However, all OPEs detected in the chlorinated group were also present in the nonchlorinated group.

Among the detected organic additives, seven were classified as rank 1, exhibiting continuous concentration increases throughout the experimental period (Figure 1b). By pipe material, five originated from PE (TCPP, TDCP, TEHP, BPF, BPAF), four from PVC (BPP, TEHP, BPA, BPAF), and only one from PPR (TEHP), suggesting lower leaching potential from PPR compared to PVC and PE. Notably, the two highest leached concentrations were both from PE: 700 ng/L TCPP in the chlorinated group and 200 ng/L BPF in the nonchlorinated group. Within the scope of the two monitored classes of plastic additives, PE exhibited a higher number and concentration of leached compounds, indicating a potentially greater chemical risk compared to the other tested materials. Substances classified as rank 2 and 3 exhibited significant concentration fluctuations, likely due to complex transformation processes such as surface adsorption, chlorination reactions, and microbial transformations (Figure S3). For rank 4, the absence of BPAP, BPZ, and TPP may be attributed to a lack of initial presence in plastic material, limited leaching potential, or concentrations falling below the detection limit.

### 3.2 Nontargeted Additives Leaching Characterized by Fluorescence EEMs

As shown in Figure S2, under chlorinated conditions, the concentration of DOM leached from experimental materials (PE, PVC, PPR) was consistently higher than that from the control material (glass). In contrast, under nonchlorinated conditions, the DOM concentration in the experimental group was significantly lower than in the control group. This suggests that microbial growth and their metabolism of DOM in nonchlorinated conditions exceeded the leaching rate of DOM from plastic pipe materials. The observed microbial metabolism is supported by the cell growth dynamics measured.

Based on fluorescence EEMs, the relative concentrations of four components (C1–C4, Figure 2a) were quantified using *F*_max_ values (Figure 2b). The mean C2 concentrations were as follows: chlorinated conditions: glass-0.023, PVC-0.033, PPR-0.047, PE-0.038; Nonchlorinated conditions: glass-0.045, PVC-0.054, PPR-0.085, PE-0.054. Regardless of chlorination, C2 concentrations from experimental groups (i.e., PVC, PPR, PE) were significantly higher than those in the control group (i.e., glass, *p* < 0.01), suggesting that the DOM leached from plastic materials exhibited spectral characteristics similar to marine or UV–visible humic-like substances. The mean C4 concentrations were: chlorinated conditions: glass-0.0075, PVC-0.016, PPR-0.020, PE-0.036; nonchlorinated conditions: glass-0.027, PVC-0.037, PPR- 0.053, PE-0.054. Similarly, C4 concentrations were significantly elevated in most plastic pipe groups compared to the control group, except in nonchlorinated PVC. This further indicates that leached DOM may also include amino acids or protein-like substances. Notably, C2 and C4 levels varied across plastic materials: C2 was the highest in PPR pipes, while C4 was most abundant in PE pipes, reflecting differences in DOM composition and leaching behavior among plastic materials. For the same pipe material, the concentration differences between chlorinated and nonchlorinated groups may be attributed to chlorine’s influence on leaching processes or variations in microbial abundance and community composition, which could affect organic matter transformation. C1 and C3 did not show consistent or significant increases in experimental groups compared to the control, suggesting minimal influence from plastic leaching and likely presence as stable components in the drinking water.

**Figure 2:**
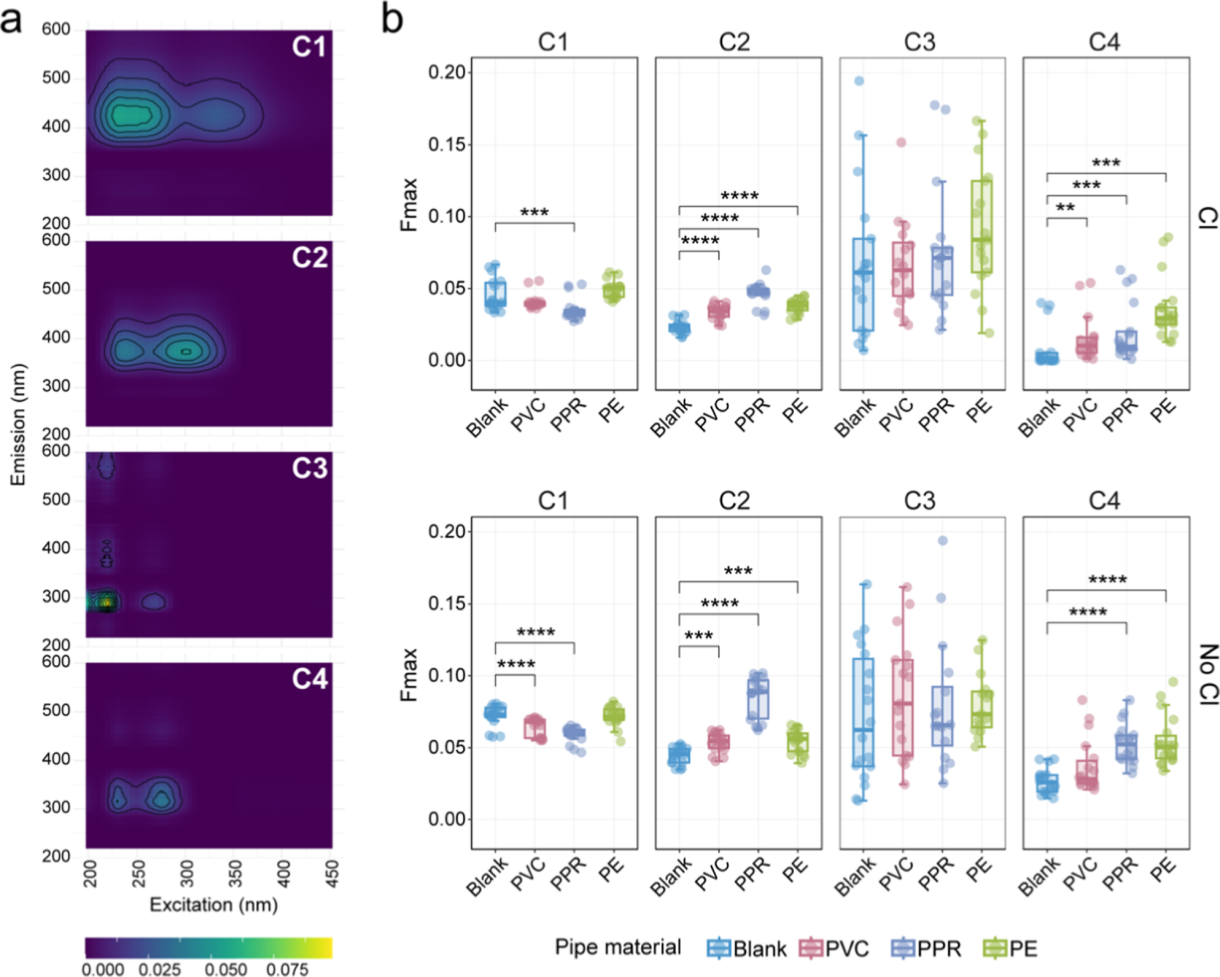
Fluorescence characterization of leached DOM from plastic pipe materials. (a) Illustration of four fluorescent components (C1, C2, C3, and C4). C1 is a humic-like component of terrestrial origin and is ubiquitous across a wide range of environments;^54,55^ C2 is marine/UV–visible humic-like, associated with phytoplankton degradation or anthropogenic sources,^56^ and similar to the humic-like component in drinking water treatments;^57^ C3 is similar to tryptophan-like component;^58,59^ C4 is similar to amino acids or proteins, associated with biological production.^60,61^ (b) Box-whisker plots of *F*_max_ values of the DOM from each treatment group (*n* = 18). Two-tailed Wilcoxon rank-sum test for comparing significant differences in fluorescent components. Significance: **, *P* < 0.01; ***, *P* < 0.001; ****, *P* < 0.0001.

### 3.3 Molecular Characterization of Newly Introduced DOM from Plastic Pipes

The newly introduced DOM molecules leached from plastic, uniquely present in the research group at day 30 but absent in both the control group and day 0, were characterized using FT-ICR MS and are shown in Figure 3a. The VK diagrams revealed that both pipe material and chlorination significantly influenced the characteristics of DOM molecules. Regarding chlorination, molecules leached under chlorinated conditions predominantly occupied the upper-left region of the VK diagrams (higher H/C, lower O/C), whereas those leached under nonchlorinated conditions clustered in the central zone (moderate H/C and O/C). In terms of pipe materials, the number of newly introduced DOM molecules leached varied: PVC leached 90 (chlorinated) to 201 (nonchlorinated) molecules, PPR leached 128 (chlorinated) to 374 (nonchlorinated), and PE leached 229 (chlorinated) to 207 (nonchlorinated). Chlorination reduced the number of newly introduced DOM molecules from PVC and PPR by 2- to 3-fold, while its effect on PE was minimal (±10%). Among the tested materials, PE under chlorination leached more diverse DOM molecules, whereas PPR without chlorination released a greater variety of DOM molecules overall.

**Figure 3:**
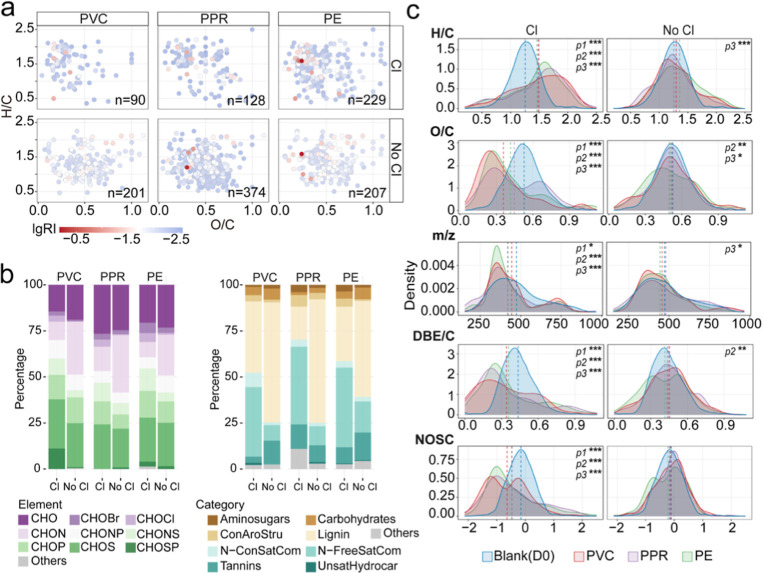
Molecular characterization of newly introduced DOM leached from plastic pipes. (a) Van Krevelen (VK) diagrams showing the distribution of DOM molecules leached from plastic pipes; (b) elemental composition (left) and compound categories (right) of leached DOM molecules under chlorinated and nonchlorinated conditions. (c) Density plots illustrating the distribution and mean values of DOM molecules across key molecular traits, including H/C ratio, O/C ratio, m/z, DBE/C, and NOSC. Statistical significance of differences between plastic-leached DOM and initial DOM (day 0) was assessed using a two-tailed Wilcoxon rank-sum test. Significance levels: *, *P* < 0.05; **, *P* < 0.01; ***, *P* < 0.001. P1, P2, and P3 indicate the significance of comparisons between PVC, PPR, and PE groups with the D0 group, respectively.

In terms of elemental composition (Figure 3b), the leached DOM in chlorinated groups was primarily composed of CHOS (24.0%–26.7%), CHO (14.4%–26.6%), and CHOP (12.5%–14.4%) in descending order. In contrast, the nonchlorinated groups were dominated by CHON (22.2%–31.3%), CHO (18.9%–24.6%), and CHOS (21.1%–23.9%). Among different plastic materials, the dominant elemental composition of leached DOM varied. In the chlorinated group, PVC was rich in CHOS (26.7%), PPR in CHO (26.6%), and PE in CHOS (24.0%). In the nonchlorinated group, PVC exhibited the highest proportion of CHON (28.9%), PPR also had CHON dominance (31.3%), while PE contained comparable levels of CHOS (23.7%).

Regarding compound composition, chlorinated groups were mainly dominated by N-free saturated compounds (37.8% in PVC to 43.2% in PE) and Lignin (18.0% in PPR to 38.9% in PVC). Although the same compound categories prevailed in the nonchlorinated groups, their proportions differed significantly. Lignin content increased substantially, ranging from 52.2% in PE to 67.1% in PPR, while N-free saturated compounds decreased to 8.5% in PVC and 16.9% in PE. Additionally, the nonchlorinated groups contained a higher proportion of Tannins, ranging from 9.1% in PPR to 15.0% in PE. Though the dominant elemental and compound compositions of leached DOM remained generally similar, the specific variations in proportions highlight the significant impact of both chlorination and pipe material on the composition of newly introduced DOM molecules.

Furthermore, in terms of chemical properties (Figure 3c), the DOM molecules leached from chlorinated plastic pipes exhibited significantly higher H/C ratios, along with lower O/C ratios, *m*/*z* values, DBE/C ratios, and NOSC compared to the intrinsic DOM in drinking water (*p* < 0.05). However, these differences were less pronounced in the nonchlorinated groups. Notably, these trends remained consistent across different pipe materials, suggesting that plastic composition had a minimal impact on the aforementioned chemical properties of the leached DOM molecules.

### 3.4 Microbial Exposure Risks Triggered by DOM Leached from Plastic Pipes

We further investigated the impact of plastic leachates on microbial exposure risks under stagnant water conditions. Chlorination had a significant impact on biomass, regardless of pipe material (Figure S2), consistently maintaining low total cell counts (TCC) throughout the experimental period (<3.0 × 10^3^ cells/ml). In contrast, under nonchlorinated conditions, TCC levels in plastic groups were significantly higher than those in control groups (glass), with control groups remaining comparable to chlorinated conditions. This suggests that DOM leaching from plastic materials significantly promoted microbial growth. Among the plastic materials, the highest TCC values were observed in PVC (2.2 × 10^4^ cells/ml), followed by PE (1.7 × 10^4^ cells/ml) and PPR (1.1 × 10^4^ cells/ml), indicating that PVC poses a higher microbial growth risk than PE and PPR.

Under both chlorinated and nonchlorinated conditions, microbial community richness significantly decreased by day 30 compared to day 0 (Figure S5a). Specifically, the richness values were: day 0–39778; chlorinated: glass-16601, PVC-12220, PPR-19722, PE-12006; Nonchlorinated: glass-24327, PVC-23156, PPR-27329, PE-26421. Notably, PPR exhibited higher richness and Shannon diversity under chlorinated conditions, whereas no clear effects under nonchlorinated conditions. This suggests that DOM leached from PPR promoted a more diverse microbial community under chlorinated conditions, potentially favoring chlorine-resistant bacteria, as supported by community composition data (Figure S6).

The microbial risks associated with plastic-leached DOM were further evaluated in terms of antibiotic resistance genes (ARGs), (opportunistic) pathogens (OPs), and virulence factors (VFs). For ARGs (Figure 4a,b), a total of 125 subtypes spanning six resistance mechanisms were identified. Under chlorinated conditions, PPR exhibited a higher richness and relative abundance of ARGs, whereas PVC and PE had no significant impact. In contrast, under nonchlorinated conditions, all plastic materials significantly increased ARG richness (by 2-fold) and relative abundance (by 2.5- to 5-fold). Among them, PVC had the most pronounced effect, elevating ARG relative abundance by 5-fold compared to the control group. Regarding resistance mechanisms, antibiotic efflux and antibiotic inactivation were significantly enriched across all groups, while antibiotic target alteration and antibiotic target protection were specifically enhanced in PVC-exposed conditions.

**Figure 4:**
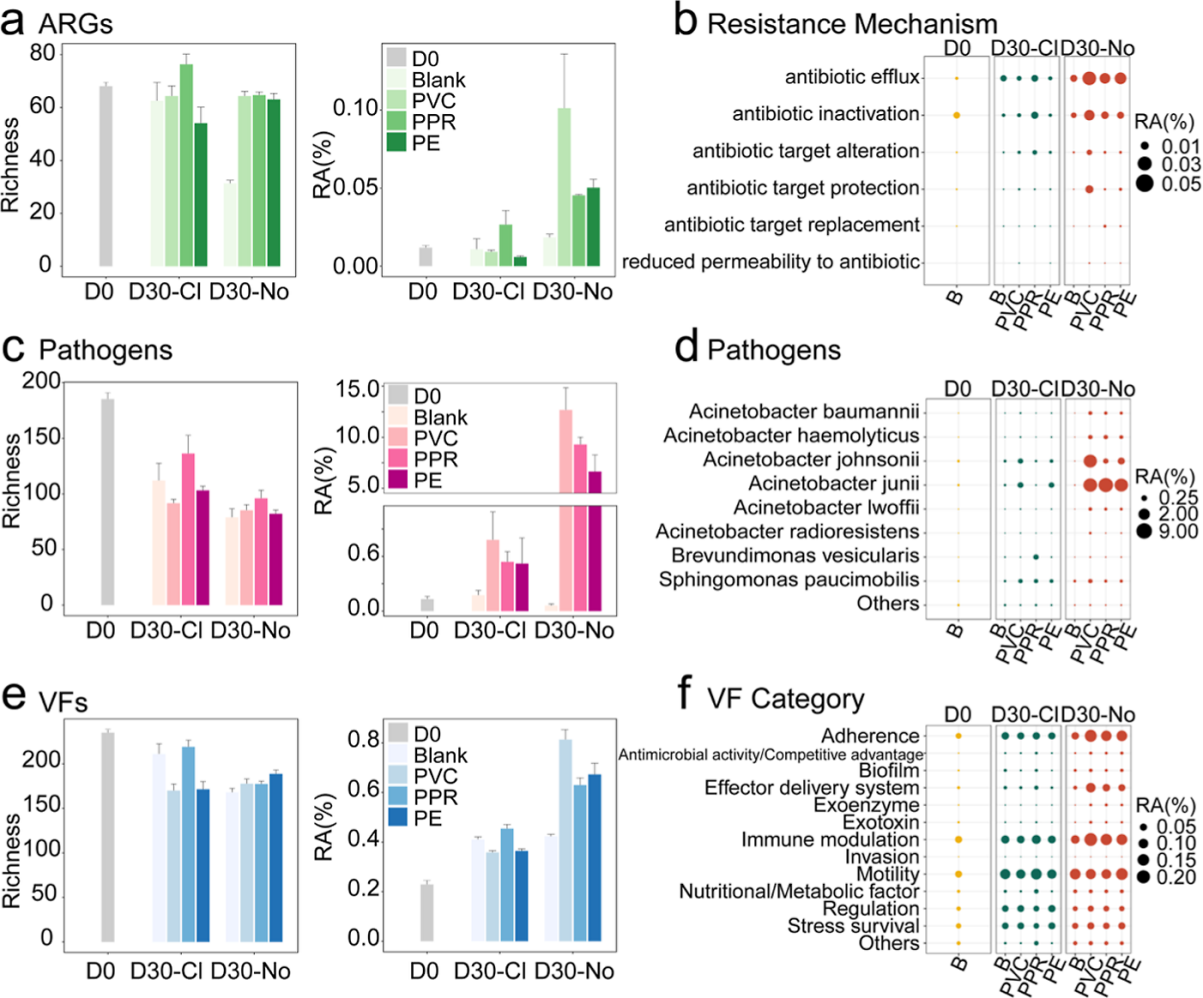
Microbial exposure risks triggered by plastic leached DOM. Bar plot of the richness and relative abundance (RA) of detected antibiotic resistance genes (ARGs) subtypes (a), pathogens (c) and virulence factors (VFs) subtypes (e). Richness refers to the number of distinct microbial taxa identified at the species level in each sample. Relative abundance refers to the percentage obtained by normalizing TPM values within each sample. Error bars represent means ± standard error (SE) (*n* = 3). Bubble plots display the relative abundance of resistance mechanisms related to ARGs (b), enriched pathogens (d) and VF categories (f). Those in bubble plots (b,f) with a relative abundance lower than 0.01% in all samples are grouped as “others”.

A total of 269 opportunistic pathogens (OPs) from 12 phyla were detected (Table S8). Although the overall number of OPs decreased, their relative abundances increased across all conditions, regardless of chlorination or pipe material (Figure 4c,d). Eight shared pathogens were promoted by leached DOM from plastic materials (Figure S7), with *Acinetobacter johnsonii*, and *Acinetobacter junii* exhibiting higher relative abundances than *Acinetobacter baumannii*, *Acinetobacter hemolyticus*, *Acinetobacter lwoffii*, *Acinetobacter radioresistans*, *Brevundimonas vesicularis*, and *Sphingomonas paucimobilis*. However, the level of enrichment varied significantly: under chlorinated conditions, OPs levels increased 5- to 7-fold, whereas under nonchlorinated conditions, they surged by hundreds- to thousands-fold. For example, *A. junii* increased from 0.001% to 5.8% (PE) −8.7% (PPR), while *A. johnsonii* rose from 0.0002% to 0.3% (PPR) −5.5% (PVC) under nonchlorinated conditions. The effects of pipe materials also varied significantly, influencing both OP abundance and diversity (Figure S8). In terms of OPs abundance, the ranking was PVC > PPR > PE. However, PPR supported the highest diversity of OPs (16–31).

A total of 309 VF subtypes were identified using the VFDB core database and classified into 13 primary VF types (Figure 4e). Similar to OPs, VF richness declined from day 0 to day 30, while the total relative abundance of VFs increased over time. Notably, under chlorinated conditions, there was no significant difference in VF relative abundance between the experimental and control groups. However, under nonchlorinated conditions, the plastic groups exhibited VF abundances 1.5–2 times higher than the control group (0.4%, glass; and 0.6% PPR −0.8% PVC). Among VF types, those showing significant increases included adherence, the effector delivery system and immune modulation factors (Figure 4f).

### 3.5 Co-Occurrence of Plastic Leached DOM and the Enriched OPs

The co-occurrence network analysis between plastic-leached DOM molecules and enriched OPs identified 198 significantly correlated molecules under chlorinated conditions and 171 under nonchlorinated conditions (|ρ| ≥ 0.7, *P* < 0.05), with only seven molecules shared between the two. The nonchlorinated network exhibited higher modularity (modularity > 0.4) than the chlorinated network (modularity < 0.4). Notably, *S. paucimobilis* dominated the largest number of DOM molecular nodes under both conditions, accounting for 95% (*n* = 189) in chlorinated environments and 33% (*n* = 56) in nonchlorinated environments (Figure 5, module 1). This suggests that *S. paucimobilis* functions as a generalist, capable of utilizing a broad spectrum of DOM, particularly under chlorinated conditions. In contrast, other OPs exhibited limited DOM associations under chlorinated conditions (*n* = 9), whereas their associations increased more than 10-fold in nonchlorinated environments (*n* = 115), indicating more diverse microbial metabolism in the absence of chlorination. Additionally, the network analysis underscored the critical role of CHO and CHOS molecules, as well as lignin and N-Free saturated compounds, in the enrichment of OPs.

**Figure 5:**
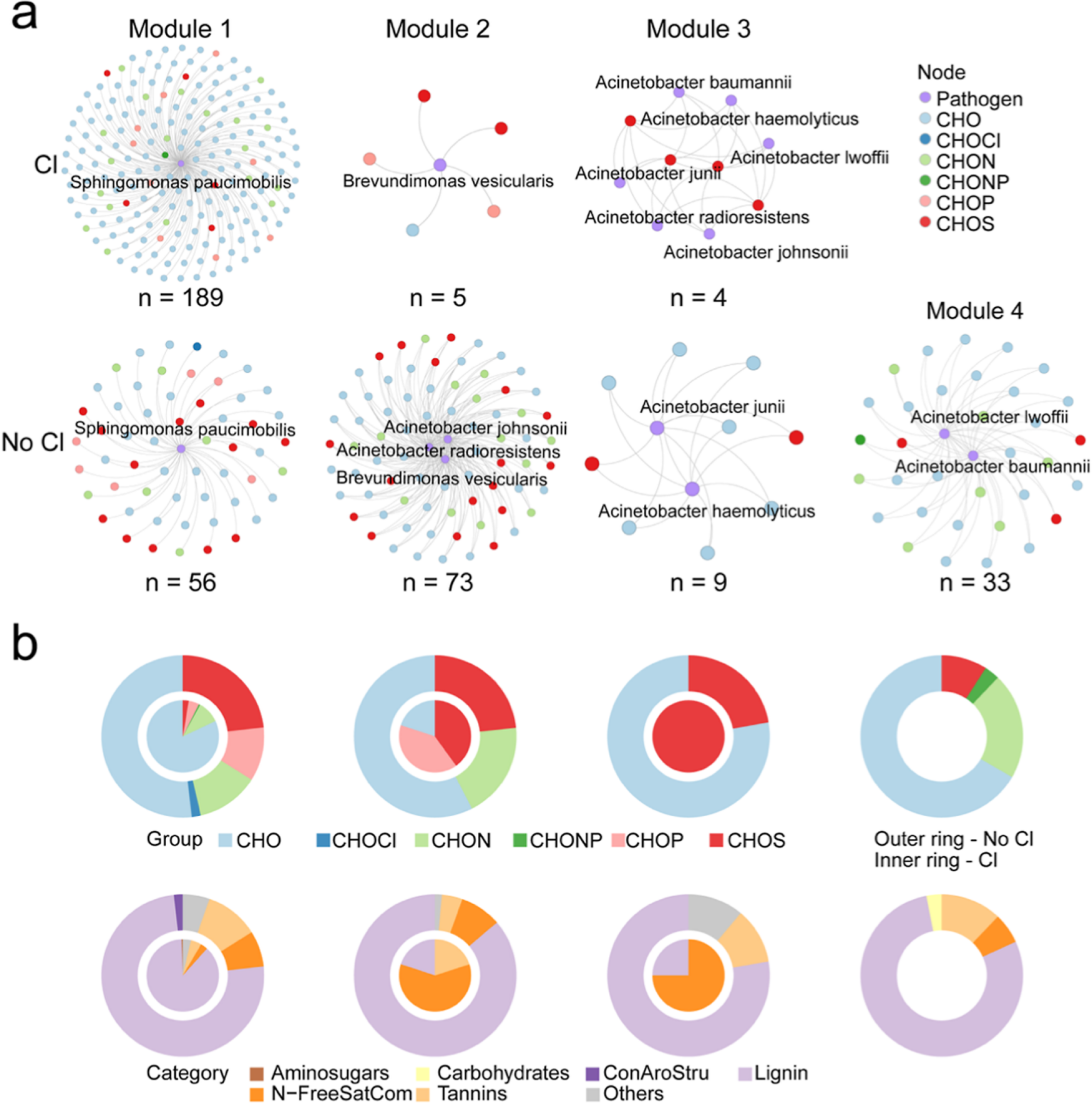
Co-occurrence network between plastic-enriched opportunistic pathogens and DOM molecules. (a) The topology of the networks. Network nodes represent DOM molecules or pathogens. The types of nodes are shown in different colors. (b) Composition of DOM molecules in different modules.

## 4 Discussion

By integrating advancements in analytical chemistry and molecular microbiology, this study explores the chemical leaching and microbial exposure risks associated with plastic pipes in drinking water systems. Expanding on previous research, it reveals the complex dynamics of organic additives and DOM leached from the plastic pipes—both spectrally and molecularly—in chlorinated and nonchlorinated water over time. Furthermore, it demonstrates that plastic pipes trigger microbial exposure risks in drinking water. These findings provide a deeper understanding of the potential hazards posed by plastic pipes in drinking water distribution systems (DWDSs) and offer critical insights for risk assessment and management.

### 4.1 Chemical Leaching from Plastic Pipes

Our study observed the continuous leaching of 13 out of 16 targeted additives over 30 days, including five BPs and eight OPEs. This aligns with previous research showing the release of organic additives from plastics into aquatic environments such as seawater, rivers, and lakes.^36,62^ While one study has examined human exposure to OPEs via water dispensers, research on additive leaching from drinking water distribution pipes remains scarce, despite the increasing use of plastic pipes and associated health risks.^63–65^ Chlorination significantly influenced the leaching of both BPs and OPEs. Specifically, chlorination reduced the concentrations of several BPs (e.g., BPF, BPA, BPAF), likely due to their transformation into chlorinated byproducts.^66,67^ Conversely, nonchlorinated conditions led to lower levels of certain OPEs (e.g., TCPP, TDCP), suggesting biodegradation.^68^ These transformation products may pose even greater health risks,^69–72^ yet they remain largely unregulated and unexplored.^73,74^ Notably, the highest concentrations of both TCPP (700 ng/L) under chlorinated conditions and BPF (200 ng/L) under nonchlorinated conditions were observed in PE pipes. Notably, BPA has not been detected in PE pipes, which is consistent with previous studies. In contrast, BPA is commonly used as an antioxidant in PVC materials,^75,76^ whereas studies on PE pipes have not reported BPA leaching.^77^ BPAP, BPZ, and TPP were not detected in any of the three pipe materials. Due to limited information on pipe formulations and leaching, it is unclear whether these compounds were absent or below detection limits.

Beyond targeted additives, nontargeted analysis confirmed significant DOM leaching, with distinct variations across pipe materials and treatment conditions.^9,78^ Under nonchlorinated conditions, we observed lower DOC levels. While previous studies have suggested that chlorination may stimulate organic leaching,^79,80^ we propose that microbial consumption played a dominant role in reducing DOC in nonchlorinated conditions. Molecular-level characterization further revealed that the number of newly introduced DOM molecules in PVC and PPR pipes was substantially higher under nonchlorinated conditions compared to the chlorinated conditions. This may be attributed to the greater microbial abundance and diversity in nonchlorinated conditions, which could promote the formation of more biotransformation products and thereby increase the number of DOM molecules.

PVC, PPR, and PE exhibited distinct DOM leaching patterns based on composition and chlorination effects. Under chlorinated conditions, PVC and PE leached more CHOS, while PPR favored CHO. In nonchlorinated conditions, CHON was more prevalent, especially in PVC and PPR. Regarding compound composition, PE consistently leached high levels of lignin, while PVC and PPR favored N-free saturated compounds under chlorinated conditions. The observed differences in N-containing DOM between chlorinated treatments may be attributed to microbial abundance. Microbially associated metabolites are predominantly N-containing compounds, including oligopeptides, amino acids, and purines. Previous studies have also reported correlations between microbial activity and the abundance of N-containing DOM molecules.^81,82^ Without chlorination, lignin and tannin leaching were more pronounced, particularly in PPR and PE. While chlorination significantly alters the leached DOM composition, the fundamental chemical property trends remain consistent across plastic types. The vast diversity of unidentified DOM molecules and their variations due to chlorination and biotransformation suggest high uncertainties and potential risks. A more comprehensive examination of plastic leachates is urgently needed to fully assess the hazards posed by plastic pipes in drinking water systems.

### 4.2 Cascaded Microbial Exposure Risks Triggered by Plastic Leachates

We found that DOM leached from plastic materials significantly increased microbial exposure risks in drinking water, particularly in the absence of chlorine disinfection. This impact was evident in elevated biomass (10–20-fold), antibiotic resistance genes (ARGs, 2.5–5-fold), opportunistic pathogens (OPs, enrichment of eight species), and virulence factors (VFs, 1.5–2-fold). Our findings align with previous studies in marine environments, demonstrating that plastic leachates stimulate microbial activity, alter community composition, and promote ARGs and VFs.^29,30,83,84^ Notably, virulence types associated with adherence were significantly enriched in plastic groups, suggesting that plastic surfaces may facilitate biofilm formation or accelerate this process via DOM leaching.^85^ This is consistent with previous findings that *S. paucimobilis*, a common drinking water bacterium, exhibited higher biofilm-forming ability on plastic pipe materials compared to metal (Al, Cu) and rubber surfaces.^86^ While microbial diversity declined over time due to competition and selective pressures,^87^ the relative abundance of specific OPs increased significantly, regardless of chlorination. This phenomenon may be attributed either to certain DOM components that promote OP growth or to the elevated tolerance of OPs to leached plastic additives compared to other microbes. For instance, *S. paucimobilis*, *A. johnsonii*, and *A. junii*—all opportunistic pathogens linked to blood-related infections—exhibited substantial enrichment.^88–90^ In nonchlorinated conditions, the abundance of *A. johnsonii*, and *A. junii* even increased by three to 4 orders of magnitude, highlighting serious risks in unchlorinated systems and under conditions of absent or depleted chlorine, such as dead-end pipes, prolonged stagnation, and water main breaks or leaks. Moreover, *S. paucimobilis* is a generalist capable of metabolizing a wide range of compounds^91,92^ and possesses strong biofilm-forming ability,^86,93^ underscoring the multifaceted microbial risks posed by plastic leaching.

The type of plastic material significantly influenced the cascading microbial risks triggered by DOM leaching. While chlorination effectively controlled biomass across all pipe types, plastic pipes—particularly PVC—promoted microbial growth in its absence, followed by PE and PPR. The pronounced microbial growth associated with PVC aligns with previous studies on bacterial regrowth potential and biofilm formation in different plastics.^94,95^ Although microbial diversity generally declined over time, PPR maintained higher richness under chlorination, likely fostering chlorine-resistant bacteria.^96^ Under nonchlorinated conditions, ARGs significantly increased across all plastic groups, with PVC having the strongest impact. OPs and VFs also exhibited an overall rise in relative abundance, with PVC supporting the highest OP levels and PPR fostering the greatest diversity. This is consistent with previous observations in marine environments, where PVC leachates were shown to alter and enrich ARGs and VFs in bacterial communities.^83^ While chlorination mitigated some microbial risks, it still led to increased OP levels. Overall, plastic-leached DOM heightened microbial risks, with PVC posing the highest risk, followed by PPR and PE.

### 4.3 Implications and Outlook

This study systematically reveals, for the first time, the dual threat plastic pipes pose to drinking water systems by integrating chemical and microbial perspectives—the release of chemical pollutants and the cascaded microbial risks. While traditional research has primarily focused on the migration of known plastic additives,^97,98^ our findings demonstrate that plastic pipes not only continuously release organic additives (e.g., BPA, TEHP) but also reshape the chemical composition of drinking water through DOM leaching. This process directly drives microbial community shifts and enriches ARGs, OPs, and VFs. These findings challenge traditional methodologies employed in the assessment of drinking water safety, which frequently distinguishes between “chemical contamination” and “microbial risk”. Thus, these insights can serve as a basis for establishing a multidimensional risk assessment framework. Furthermore, it provides novel insights for assessing the worldwide trends in substituting metal pipes with various types of plastic pipes.

The double-edged sword effect of chlorine disinfection further complicates these risks. While chlorine effectively controls microbial growth, its strong oxidative properties accelerate plastic aging, enhancing additive release and promoting the formation of potentially more toxic chlorinated byproducts (e.g., Cl-BPA).^99,100^ Moreover, the unique molecular characteristics of newly introduced DOM may introduce novel toxicity concerns. Conversely, in the absence of chlorine disinfection, plastic-derived DOM significantly elevates microbial risks, and the degradation products of parent pollutants (e.g., OPE metabolites) may exhibit greater ecotoxicity. These findings highlight the limitations of current water quality standards, which primarily focus on parent pollutant concentrations, and underscore the urgent need to incorporate chlorinated byproducts, microbial degradation products, and cumulative toxicity effects into drinking water risk assessments.

This study employed a laboratory-based static simulation system, effectively isolating environmental interference to clarify the core mechanisms of plastic pipe impacts. However, this study only represents scenarios of water stagnation in plastic pipes. Under flow conditions, hydraulic shear stress may influence the leaching behavior of plastic additives. In addition, increased water flow may suppress microbial growth and modify the interactions between microbes and plastic-leached compounds. This study also focused only on plastic additives and DOM in the aqueous phase, without considering the potential adsorption onto particles. Therefore, future research should further investigate the role of particulate matter to provide a more comprehensive understanding of the leaching dynamics of plastics. Additionally, while metagenomic analysis provides insights into the genetic potential of microbial risks, further validation through transcriptomic and metabolomic approaches is needed to assess gene expression activity and transmission efficiency. Real DWDSs are more complex, with biofilm attached to pipe surfaces as well as accumulated sediments. These dense microbial communities may accelerate the aging of plastic pipes and further influence the transformation of leached chemical compounds. A longer term of experiment is necessary to acquire a closer-to-practice result. Future research should integrate multiomics analyses and dynamic distribution system simulations to comprehensively evaluate the longer-term impacts of plastic pipes on drinking water safety.

## Supplementary Material



## Data Availability

Sequence data associated with this project have been deposited in the NCBI Short Read Archive database (Accession Number: PRJNA1230761). Plastic pipes continuously leach diverse dissolved organic molecules into drinking water, not only posing chemical risks, but also accelerating microbial growth and enriching antibiotic resistance genes, opportunistic pathogens, and virulence factors.

## Notes

Liquid chromatography and FT-ICR MS conditions, formula assignment and parameter calculations (text S1–S3); standards and water quality, identification and analysis of organic additives, formula categorization, target additives in control, human pathogenic species and pathogen abundance, DOM molecule co-occurrence networks (table S1–S9); additional graphs on experimental schematic, biomass and DOC, organic additives concentration, microbial diversity and composition, pathogens, etc. (figure S1–S9) (PDF)
