# Supplementary material for: Unveiling Chemical-Microbial Cascade Risk Factors from Plastic Pipe Leaching in Drinking Water
Source: Environ Sci Technol. 2025 Oct 14;59(42):22887–900. doi: 10.1021/acs.est.5c10244 (PMC12573800; doi:10.1021/acs.est.5c10244)
Supplement: Supplementary file 1 [file es5c10244_si_001.pdf]

Supplementary Information for:

Unveiling Chemical-Microbial Cascade Risk Factors from Plastic Pipe Leaching in Drinking Water

Mengqing Fan [1, 2], Ziqian Wang [1, 2], Mingchen Yao [1, 2], Xiaoming Li [1], Walter van der Meer [3], Yu Tao [4], Joan B. Rose [5], Gang Liu [1, 2, 6 *]

[1] *Key Lab of Aquatic Chemistry, State Key Lab of Regional Environment, Research Centre for Eco-Environmental Sciences, Chinese Academy of Sciences, Beijing, 100085, China*

[2] *University of Chinese Academy of Sciences, Beijing, 100049, China*

[3] *Science and Technology, University of Twente, P.O. Box 217, 7500AE Enschede, the Netherlands*

[4] *School of Eco-Environment, Harbin Institute of Technology, Shenzhen, 518055, China*

[5] *Department of Fisheries and Wildlife, Michigan State University, East Lansing, MI, 48823, USA*

[6] *Sanitary engineering, Department of Water management, Faculty of Civil Engineering and Geosciences, Delft University of Technology, P.O. Box 5048, 2600 GA Delft, the Netherlands*

***Corresponding author:**

Prof. Dr Gang Liu,
Research Center for Eco-Environmental Sciences,
Chinese Academy of Sciences,
Beijing, China
email: gliu@rcees.ac.cn; g.liu-1@tudelft.nl

Pages: 34

Text: 3

Table: 9

Figure: 9

Text S1. *The detailed liquid chromatography conditions of BPs and OPEs.*

BPs

The separation of BPs was achieved using a XTerra MS C18 column (2.1 mm × 150 mm, 5 μm particle size, Waters, USA). Ultrapure water (A) and methanol (B) were used as the mobile phases, the column oven was 25°C, the injection volume was 20μL, and the flow rate was 400 μL/min. The gradient was set as follows: the initial 40% B in 0.5 min; followed by an increase to 100% B in 1 min and held for 2 min. Finally, the gradient was returned to the initial conditions of 40% B in 0.1 min and held for 2.4 min. The MS was operated in negative ion multiple reaction-monitoring mode (MRM).

OPEs

The separation of OPEs was achieved using a Kinetex® 2.6 μm Biphenyl 100 Å LC Column (3.0 mm × 100 mm, Phenomenex, USA). 0.1% formic acid in water (A) and methanol (B) were used as the mobile phases, the column oven was 25°C, the injection volume was 10μL, and the flow rate was 400 μL/min. The gradient was set as follows: the initial 30% B and held for 0.5 min; then increased linearly to 70% in 0.5 min; followed by an increase to 95% B in 3 min; then increased to 100% B in 4 min and held for 1 min. Finally, the gradient was returned to the initial conditions of 30% B in 1 min and held for 5 min. The MS was operated in positive ion multiple reaction-monitoring mode (MRM).

Text S2. *Analytical conditions of FT-ICR MS*

The samples were stored in 2 mL amber vials (Agilent, USA) at -20°C until FT-ICR MS analysis. FT-ICR MS measurements were performed within three days of the end of the experiment. The injection mode was continuous, the injection rate was 120 μL/h, the capillary inlet voltage was -4.0 kV, the ion accumulation time was 0.06 s, and the mass range was 100-1600 Da. The instrument was calibrated with 10 mmol/L sodium formate before the detection of the samples, and the internal standard was calibrated with the soluble organic matter (with a known molecular formula) after the detection of the samples. The instrument was calibrated with 10 mmol/L sodium formate before sample detection, and with soluble organic matter (known molecular formula) after sample detection. After calibration, the mass errors were less than 1 ppm.

Text S3. *Formula assignment conditions and molecular parameters calculation.*

According to Fu et al. [1], Non-halogenated and halogenated formulae were assigned to signal-to-noise ratio (S/N) ≥ 6 and 10, respectively with the following calculation conditions: (1) mass error 1.0 ppm (relative to exact mass value); (2) an integer double bond equivalent (DBE) (≥ 0); (3) 0.3≤ (H+Cl+Br)/C ≤ 2.25 and 0< O/C ≤ 1.2 for formulae with C≥ 5; 0.3≤ (H+Cl+Br)/C ≤ 4 and 0≤ O/C ≤ 1.2 for formulae with 1≤ C≤ 4; (4) 1≤^{12}C≤50 and ^{13}C ≤ 3; (5) -14 ≤ DBE-O ≤ 14 (solutions with -10 ≤ DBE-O ≤ 10 were preferably selected); (6) 0≤ ^{18}O ≤ 1; (7) 0≤ ^{14}N ≤ 5 and 0≤ ^{15}N ≤ 1; (8) 0≤ ^{32}S ≤ 3, 0≤ ^{33}S ≤ 1, and 0≤ ^{34}S ≤ 1; (9) 0≤ P ≤ 1; (10) 0≤ ^{35}Cl ≤ 5 and 0≤ ^{37}Cl ≤ 5; (11) 0≤ ^{79}Br ≤ 5 and 0≤ ^{81}Br ≤ 5.

The double bond equivalence (DBE), the modified aromaticity index (AI_{mod}) and the nominal oxidation state of carbon (NOSC) of each compound were calculated as follows:

$$DBE = 1 + C - \frac{(H + Cl + Br + F - N - P)}{2} \quad (1)$$

$$AI_{mod} = \frac{DBE_{AI}}{C_{AI}} = \frac{1 + C - 0.5O - S - 0.5(N + H + P + Cl + Br + F)}{C - 0.5O - S - N - P} \quad (2)$$

$$NOSC = 4 - \frac{(4nC + nH - 3nN - 2nO - 2nS + 5nP - nCl - nBr - nF)}{nC} \quad (3)$$

where *n*C, *n*H, *n*O, *n*N, *n*S, *n*P, *n*Cl, *n*Br, and *n*F refer to the stoichiometric number of C, H, O, N, S, P, Cl, Br, and F atoms per formula, respectively. For **Equation 2**, if $DBE_{AI} \leq 0$ or $C_{AI} \leq 0$, then $AI_{mod} = 0$.

Table S1. Regulatory Standards for Hygiene and Safety of Drinking Water Pipes.

	China	USA	Netherlands
PVC<br>PPR<br>PE	GB/T 17219	NSF/ANSI 61	EN 12873

Table S2. *Basic water quality information.*[a]

Parameter	Value (mean ± SD)
Temperature (°C)	24.5 ± 0.6
pH	7.30 ± 0.10
DO (mg/L)	7.91 ± 0.89
EC (μS/cm)	298 ± 33
TN (mg/L)	1.30 ± 0.20
TP (mg/L)	0.08 ± 0.01
TOC (mg/L)	3.71 ± 0.43
TCC (cells/L)	988 ± 29
ATP (ng/L)	1.43 ± 0.97

Table S3. *Identification and physico-chemical properties of target additives.*

Acronym	Compound	CAS number	MW	Log Kow
**Organophosphorus esters (OPEs)**				
TPP	Tripropyl phosphate	513-08-6	224.1	1.9
TiBP	Triisobutyl phosphate	126-71-6	266.2	3.5
TnBP	Tri-n-butyl phosphate	126-73-8	266.2	4.0
TCEP	Tris (2-chloroethyl) phosphate	115-96-8	283.9	1.8
TCPP	Tris (1-chloro-2-propyl) phospahte	13674-84-5	326.0	2.6
TDCP	Tris(1,3-dichloro-2-propyl] phosphate	13674-87-8	427.9	3.7
TPhP	Triphenyl phosphate	115-86-6	326.1	4.6
EHDPP	2-Ethylhexyl diphenyl phosphate	1241-94-7	362.2	5.7
TEHP	Tri(2-ethylhexyl) phosphate	78-42-2	434.3	9.5
TnBP-d27	Tri-n-butyl phosphate-d27	61196-26-7	293.3	
TPhP-d15	Triphenyl phosphate-d15	1173020-30-8	341.2	
**Bisphenols (BPs)**				
BPA	bisphenol A	80-05-7	228.1	3.3
BPAF	bisphenol AF	14878-61-1	336.1	4.5
BPAP	bisphenol AP	1571-75-1	290.1	4.4
BPF	bisphenol F	620-92-8	200	2.9
BPP	bisphenol P	2167-51-3	346.2	6.1
BPS	bisphenol S	80-09-1	250	1.9
BPZ	bisphenol Z	843-55-0	268.1	5.4
BPA-d16	bisphenol A-d16	96210-87-6	244.4	

Table S4. *MS/MS parameters used for compounds analysis.*

Analytes	Internal standard (IS)	Ion mode	Quantitative transition		Qualitative transition		Retention time (min)	Recovery rate	MDLs （ng/L）
**Organophosphorus esters (OPEs)**									
TPP	TnBP-D27	+	225.1	183.2	225.1	98.9	5.29	84.99	0.37
TiBP	TnBP-D27	+	267.1	155.2	267.1	99	5.89	79.57	0.63
TnBP	TnBP-D27	+	267.1	155.2	267.1	99	6.09	106.83	0.40
TCEP	TnBP-D27	+	287	225	287	99.1	4.89	122.81	1.09
TCPP	TnBP-D27	+	329.2	99.1	327	99.1	5.59	137.86	0.77
TDCP	TPhP-D15	+	431.1	208.9	431.1	99.1	6.1	122.42	0.20
TPhP	TPhP-D15	+	327.2	152.2	327.2	77.2	6.54	104.55	1.31
EHDPP	TPhP-D15	+	363.3	251	363.3	76.9	7.1	119.72	0.09
TEHP	TPhP-D15	+	435.4	99	435.4	113	7.88	78.58	0.32
TnBP-D27	-	+	294	102	294	230	6.01		
TPhP-D15	-	+	342	223.1	342	160.2	6.48		
**Bisphenols (BPs)**									
BPA	BPA-D16	-	226.9	133	226.9	212.1	3.09	72.70	3.03
BPAF	BPA-D16	-	335	265.1	335	289.1	3.39	139.16	0.27
BPAP	BPA-D16	-	289.3	274.1	289.3	211	3.39	71.53	6.06
BPF	BPA-D16	-	198.9	93	198.9	105.1	2.89	71.50	1.38
BPP	BPA-D16	-	345.2	330.2	345.2	252.2	3.77	114.96	7.24
BPS	BPA-D16	-	248.9	108	248.9	92	2.57	136.30	0.26
BPZ	BPA-D16	-	267.1	223.1	267.1	173	3.48	97.38	27.86
BPA-D16	-	-	241.3	223.1	241.3	142	3.06		

Table S5. *Criteria of molecular formula categorization*[1].

Molecular class	Criteria
Carbohydrates	$0.71 \le O/C \le 1.2$, $1.5 \le H/C \le 2.4$
Aminosugars	$0.52 \le O/C \le 0.71$, $1.5 \le H/C \le 2.2$, $N > 0$
N-containing saturated compounds	$0 \le O/C \le 0.52$, $1.5 \le H/C \le 2.2$, $N > 0$
N-free saturated compounds	$0 \le O/C \le 0.52$, $1.5 \le H/C \le 2.2$, $N = 0$
Tannins	$0.67 \le O/C \le 1.2$, $0.5 \le H/C \le 1.5$
Lignins	$0.1 \le O/C \le 0.67$, $0.7\le H/C \le 1.5$
Unsaturated hydrocarbons	$0 \le O/C \le 0.1$, $0.7\le H/C \le 1.5$
Condensed aromatic structures	$0 \le O/C \le0.67$, $0.2 \le H/C \le 0.7$
Others	Formula beyond the above criteria

Table S6. Concentrations of detected plastic additives in the control group during the experiment.

Sample	Day0	Day15-Cl	Day15-No	Day30-Cl	Day30-No
EHDPP (ng/L)	1.62±0.36	1.66±0.16	1.33±0.05	1.55±0.05	1.39±0.03
TCEP (ng/L)	15.1±1.12	17.45±1.48	15.01±1.02	15.78±0.15	15.51±1
TCPP (ng/L)	92.43±4.45	77.48±3.38	70.15±2.89	69.54±1.29	66.15±4.5
TiBP (ng/L)	4.84±0.55	4.52±0.25	5.74±0.68	4.61±0.35	4.41±1.17
TnBP (ng/L)	2.77±0.22	2.77±0.06	3.19±0.29	2.71±0.06	2.79±0.1
TPhP (ng/L)	2.6±0.1	3±0.33	3.5±0.63	2.79±0.09	2.63±0.19

Table S7. *Human pathogenic species.*

	Human pathogenic species	
Abiotrophia defectiva	*Ehrlichia ewingii*	*Nocardia pseudobrasiliensis*
Achromobacter piechaudii	*Eikenella corrodens*	*Nocardia transvalensis*
Achromobacter xylosoxidans	*Enterobacter aerogenes*	*Ochrobactrum anthropi*
Acidaminococcus fermentans	*Enterobacter amnigenus*	*Oligella ureolytica*
Acinetobacter baumannii	*Enterobacter asburiae*	*Oligella urethralis*
Acinetobacter calcoaceticus	*Enterobacter cancerogenus*	*Orientia tsutsugamushi*
Acinetobacter haemolyticus	*Enterobacter cloacae*	*Paenibacillus alvei*
Acinetobacter johnsonii	*Enterobacter gergoviae*	*Paenibacillus macerans*
Acinetobacter junii	*Enterobacter hormaechei*	*Pantoea agglomerans*
Acinetobacter lwoffii	*Enterobacter sakazakii*	*Pasteurella aerogenes*
Acinetobacter radioresistens	*Enterococcus avium*	*Pasteurella caballi*
Actinobacillus actinomycetemcomitans	*Enterococcus casseliflavus*	*Pasteurella canis*
Actinobacillus equuli	*Enterococcus durans*	*Pasteurella dagmatis*
Actinobacillus hominis	*Enterococcus faecalis*	*Pasteurella multocida*
Actinobacillus lignieresii	*Enterococcus faecium*	*Pasteurella pneumotropica*
Actinobacillus pleuropneumoniae	*Enterococcus flavescens*	*Pasteurella stomatis*
Actinobacillus suis	*Enterococcus gallinarum*	*Peptinophilus asaccharolyticus*
Actinobacillus ureae	*Enterococcus hirae*	*Peptococcus niger*
Actinomyces georgiae	*Enterococcus mundtii*	*Peptostreptococcus anaerobius*
Actinomyces gerencseriae	*Enterococcus raffinosus*	*Photobacterium damselae*
Actinomyces israelii	*Erysipelothrix rhusiopathiae*	*Plesiomonas shigelloides*
Actinomyces meyeri	*Escherichia coli*	*Porphyromonas asaccharolytica*
Actinomyces naeslundii	*Eubacterium brachy*	*Porphyromonas catoniae*
Actinomyces neuii	*Eubacterium combesii*	*Porphyromonas circumdentaria*
Actinomyces odontolyticus	*Eubacterium contortum*	*Porphyromonas endodontalis*
Actinomyces radingae	*Eubacterium cylindroides*	*Porphyromonas gingivalis*
Actinomyces turicensis	*Eubacterium limosum*	*Porphyromonas levii*
Aerococcus viridans	*Eubacterium moniliforme*	*Porphyromonas macacae*
Aeromonas caviae	*Eubacterium multiforme*	*Prevotella bivia*
Aeromonas hydrophila	*Eubacterium nodatum*	*Prevotella buccae*
Aeromonas sobria	*Eubacterium rectale*	*Prevotella buccalis*
Aeromonas veronii	*Eubacterium saburreum*	*Prevotella corporis*
Alcaligenes odorans	*Eubacterium saphenum*	*Prevotella dentalis*
Amycolatopsis orientalis	*Eubacterium sulci*	*Prevotella denticola*
Anaerococcus lactolyticus	*Eubacterium tenue*	*Prevotella disiens*
Anaerococcus prevotii	*Ewingella americana*	*Prevotella enoeca*
Anaerococcus vaginalis	*Fibrobacter intestinalis*	*Prevotella heparinolytica*
Arcanobacterium bernardiae	*Filifactor alocis*	*Prevotella intermedia*
Arcanobacterium heamolyticum	*Finegoldia magna*	*Prevotella loescheii*

Arcanobacterium pyogenes	*Fluoribacter bozemanae*	*Prevotella melaninogenica*
Arcobacter butzleri	*Fluoribacter dumoffii*	*Prevotella nigrescens*
Arcobacter cryaerophilus	*Fluoribacter gormanii*	*Prevotella oralis*
Anaplasma phagocytophila	*Francisella tularensis*	*Prevotella oris*
Bacillus anthracis	*Fusobacterium mortiferum*	*Prevotella oulora*
Bacillus cereus	*Fusobacterium necrophorum*	*Prevotella ruminicola*
Bacillus circulans	*Fusobacterium nucleatum*	*Prevotella tannerae*
Bacillus coagulans	*Fusobacterium periodonticum*	*Prevotella veroralis*
Bacillus licheniformis	*Fusobacterium ulcerans*	*Prevotella zoogleoformans*
Bacillus mycoides	*Fusobacterium varium*	*Propionibacterium acnes*
Bacillus pumilus	*Gardnerella vaginalis*	*Propionibacterium avidum*
Bacillus sphaericus	*Gemella morbillorum*	*Propionibacterium granulosum*
Bacillus subtilis	*Gordonia amarae*	*Propionibacterium propionicus*
Bacillus thuringiensis	*Gordonia bronchialis*	*Proteus mirabilis*
Bacteroides caccae	*Gordonia rubropertincta*	*Proteus penneri*
Bacteroides distasonis	*Gordonia sputi*	*Proteus vulgaris*
Bacteroides eggerthii	*Gordonia terrae*	*Providencia alcalifaciens*
Bacteroides forsythus	*Granulicatella adjacens*	*Providencia rettgeri*
Bacteroides fragilis	*Haemophilus aphrophilus*	*Providencia stuartii*
Bacteroides galacturonicus	*Haemophilus ducreyi*	*Pseudomonas aeruginosa*
Bacteroides merdae	*Haemophilus haemolyticus*	*Pseudomonas alcaligenes*
Bacteroides ovatus	*Haemophilus influenzae*	*Pseudomonas fluorescens*
Bacteroides pectinophilus	*Haemophilus parahaemolyticus*	*Pseudomonas pseudoalcaligenes*
Bacteroides splanchnicus	*Haemophilus parainfluenzae*	*Pseudomonas putida*
Bacteroides stercoris	*Haemophilus paraphrophilus*	*Pseudomonas stutzeri*
Bacteroides thetaiotaomicron	*Haemophilus segnis*	*Pseudonocardia autotrophica*
Bacteroides uniformis	*Hafnia alvei*	*Pseudoramibacter alactolyticus*
Bacteroides ureolyticus	*Helicobacter cinaedi*	*Psychrobacter phenylpyruvicus*
Bacteroides vulgatus	*Helicobacter fennelliae*	*Rahnella aquatilis*
Bartonella bacilliformis	*Helicobacter heilmannii*	*Ralstonia pickettii*
Bartonella elizabethae	*Helicobacter pullorum*	*Rhodococcus equi*
Bartonella henselae	*Helicobacter pylori*	*Rhodococcus erythropolis*
Bartonella quintana	*Kingella denitrificans*	*Rhodococcus fascians*
Bergeyella zoohelcum	*Kingella kingae*	*Rhodococcus rhodnii*
Bifidobacterium dentium	*Klebsiella granulomatis*	*Rhodococcus rhodochrous*
Bilophila wadsworthia	*Klebsiella ornithinolytica*	*Rickettsia africae*
Bordetella avium	*Klebsiella oxytoca*	*Rickettsia akari*
Bordetella bronchiseptica	*Klebsiella pneumoniae*	*Rickettsia australis*
Bordetella parapertussis	*Kluyvera ascorbata*	*Rickettsia conorii*
Bordetella pertussis	*Kluyvera cryocrescens*	*Rickettsia felis*
Borrelia brasiliensis	*Lactobacillus sp.*	*Rickettsia honei*
Borrelia burgdorferi	*Legionella anisa*	*Rickettsia japonica*

Borrelia caucasica	*Legionella birminghamensis*	*Rickettsia massiliae*
Borrelia crocidurae	*Legionella cherrii*	*Rickettsia prowazekii*
Borrelia duttonii	*Legionella cincinnatiensis*	*Rickettsia rickettsii*
Borrelia hermsii	*Legionella feeleii*	*Rickettsia sibirica*
Borrelia hispanica	*Legionella hackeliae*	*Rickettsia typhi*
Borrelia latyschewii	*Legionella jordanis*	*Rothia dentocariosa*
Borrelia mazzottii	*Legionella lansingensis*	*Ruminococcus productus*
Borrelia parkeri	*Legionella longbeachae*	*Saccharomonospora viridis*
Borrelia persica	*Legionella oakridgensis*	*Saccharopolyspora rectivirgula*
Borrelia recurrentis	*Legionella pneumophila*	*Salmonella bongori*
Borrelia turicatae	*Legionella rubrilucens*	*Salmonella choleraesuis*
Borrelia venezuelensis	*Legionella sainthelensi*	*Salmonella enteritidis*
Brevibacillus brevis	*Legionella tucsonensis*	*Salmonella typhi*
Brevundimonas diminuta	*Legionella wadsworthii*	*Salmonella typhimurium*
Brevundimonas vesicularis	*Leifsonia aquatica*	*Sebaldella termitidis*
Brucella melitensis	*Leptospira borgpetersenii*	*Selenomonas artemidis*
Burkholderia cepacia	*Leptospira inadai*	*Selenomonas dianae*
Burkholderia mallei	*Leptospira interrogans*	*Selenomonas flueggei*
Burkholderia pseudomallei	*Leptospira kirschneri*	*Selenomonas infelix*
Campylobacter coli	*Leptospira meyeri*	*Selenomonas noxia*
Campylobacter concisus	*Leptospira noguchii*	*Serratia ficaria*
Campylobacter curvus	*Leptospira santarosai*	*Serratia marcescens*
Campylobacter fetus	*Leptospira weilii*	*Serratia odorifera*
Campylobacter gracilis	*Leptotrichia buccalis*	*Serratia plymuthica*
Campylobacter hyointestinalis	*Listeria ivanovii*	*Serratia proteamaculans*
Campylobacter jejuni	*Listeria monocytogenes*	*Serratia rubidaea*
Campylobacter lari	*Listeria seeligeri*	*Shigella boydii*
Campylobacter rectus	*Listeria welshimeri*	*Shigella dysenteriae*
Campylobacter sputorum	*Mannheimia haemolytica*	*Shigella flexneri*
Campylobacter upsaliensis	*Megamonas hypermegale*	*Shigella sonnei*
Capnocytophaga canimorsus	*Megasphaera sp.*	*Sphingomonas paucimobilis*
Capnocytophaga cynodegmi	*Micromonas micros*	*Spirillum minus*
Capnocytophaga gingivalis	*Mogibacterium timidum*	*Staphylococcus aureus*
Capnocytophaga ochracea	*Moraxella (Branhamella) catarrhalis*	*Staphylococcus epidermidis*
Capnocytophaga sputigena	*Moraxella (Branhamella) caviae*	*Staphylococcus haemolyticus*
Cardiobacterium hominis	*Moraxella (Branhamella) cuniculi*	*Staphylococcus hyicus*
Cedecea davisae	*Moraxella (Branhamella) ovis*	*Staphylococcus intermedius*
Cedecea lapagei	*Moraxella (Moxarella) atlantae*	*Staphylococcus lugdunensis*
Cedecea neteri	*Moraxella (Moxarella) bovis*	*Staphylococcus saprophyticus*
Cellulomonas cellulans	*Moraxella (Moxarella) lacunata*	*Staphylococcus warneri*
Cellulomonas turbata	*Moraxella (Moxarella) liquefaciens*	*Stenotrophomonas maltophilia*
Centipeda periodontii	*Moraxella (Moxarella) nonliquefaciens*	*Streptobacillus moniliformis*

Chlamydia trachomatis	*Moraxella (Moxarella) osloensis*	*Streptococcus acidominimus*
Chlamydophila pneumoniae	*Moraxella lincolnii*	*Streptococcus agalactiae*
Chlamydophila psittaci	*Morganella morganii*	*Streptococcus anginosus*
Chromobacterium violaceum	*Mycobacterium abscessus*	*Streptococcus bovis*
Chryseobacterium balustinum	*Mycobacterium africanum*	*Streptococcus canis*
Chryseobacterium meningosepticum	*Mycobacterium asiaticum*	*Streptococcus constellatus*
Citrobacter amalonaticus	*Mycobacterium avium*	*Streptococcus criceti*
Citrobacter braakii	*Mycobacterium bovis*	*Streptococcus equi*
Citrobacter farmeri	*Mycobacterium celatum*	*Streptococcus gordonii*
Citrobacter freundii	*Mycobacterium chelonae*	*Streptococcus intermedius*
Citrobacter koseri	*Mycobacterium conspicuum*	*Streptococcus milleri*
Citrobacter rodentium	*Mycobacterium fortuitum*	*Streptococcus mitis*
Citrobacter sedlakii	*Mycobacterium genavense*	*Streptococcus mutans*
Citrobacter werkmanii	*Mycobacterium gordonae*	*Streptococcus pneumoniae*
Citrobacter youngae	*Mycobacterium haemophilum*	*Streptococcus pyogenes*
Clostridium baratii	*Mycobacterium kansasii*	*Streptococcus salivarius*
Clostridium bifermentans	*Mycobacterium leprae*	*Streptococcus sanguis*
Clostridium botulinum	*Mycobacterium malmoense*	*Streptococcus sobrinus*
Clostridium butyricum	*Mycobacterium marinum*	*Streptococcus suis*
Clostridium chauvoei	*Mycobacterium mucogenicum*	*Streptococcus uberis*
Clostridium difficile	*Mycobacterium peregrinum*	*Sutterella wadsworthensis*
Clostridium fallax	*Mycobacterium porcinum*	*Suttonella indologenes*
Clostridium histolyticum	*Mycobacterium scrofulaceum*	*Tatlockia maceachernii*
Clostridium novyi	*Mycobacterium senegalense*	*Tatlockia micdadei*
Clostridium perfringens	*Mycobacterium shimoidei*	*Tatumella ptyseos*
Clostridium ramosum	*Mycobacterium simiae*	*Treponema carateum*
Clostridium septicum	*Mycobacterium smegmatis*	*Treponema pallidum*
Clostridium sordellii	*Mycobacterium szulgai*	*Tropheryma whippelii*
Clostridium sporogenes	*Mycobacterium tuberculosis*	*Tsukamurella inchonensis*
Clostridium tertium	*Mycobacterium ulcerans*	*Tsukamurella paurometabola*
Clostridium tetani	*Mycobacterium xenopi*	*Tsukamurella pulmonis*
Collinsella aerofaciens	*Mycoplasma fermentans*	*Tsukamurella tyrosinosolvens*
Comamonas testosteroni	*Mycoplasma genitalium*	*Ureaplasma urealyticum*
Corynebacterium afermentans	*Mycoplasma hominis*	*Veillonella atypica*
Corynebacterium amycolatum	*Mycoplasma pneumoniae*	*Veillonella dispar*
Corynebacterium argentoratense	*Mycoplasma salivarium*	*Veillonella parvula*
Corynebacterium bovis	*Myroides odoratus*	*Vibrio alginolyticus*
Corynebacterium diphtheriae	*Neisseria cinerea*	*Vibrio cholerae*
Corynebacterium jeikeium	*Neisseria elongata*	*Vibrio cincinnatiensis*
Corynebacterium kutscheri	*Neisseria flava*	*Vibrio fluvialis*
Corynebacterium macginleyi	*Neisseria flavescens*	*Vibrio furnissii*

Corynebacterium minutissimum	*Neisseria gonorrhoeae*	*Vibrio hollisae*
Corynebacterium propinquum	*Neisseria lactamica*	*Vibrio mimicus*
Corynebacterium pseudodiphthericum	*Neisseria meningitidis*	*Vibrio parahaemolyticus*
Corynebacterium pseudotuberculosis	*Neisseria mucosa*	*Vibrio vulnificus*
Corynebacterium striatum	*Neisseria perflava*	*Wolinella succinogenes*
Corynebacterium ulcerans	*Neisseria sicca*	*Yersinia bercovieri*
Corynebacterium urealyticum	*Neisseria subflava*	*Yersinia enterocolitica*
Corynebacterium xerosis	*Neisseria weaveri*	*Yersinia frederiksenii*
Coxiella burnetii	*Neorickettsia sennetsu*	*Yersinia intermedia*
Delftia acidovorans	*Nocardia asteroides*	*Yersinia kristensenii*
Dermatophilus congolensis	*Nocardia brasiliensis*	*Yersinia mollaretii*
Dichelobacter nodosus	*Nocardia caviae*	*Yersinia pestis*
Edwardsiella hoshinae	*Nocardia farcinica*	*Yersinia pseudotuberculosis*
Edwardsiella tarda	*Nocardia nova*	*Yersinia rohdei*
Eggerthella lenta	*Nocardia otitidiscaviarum*	*Yersinia ruckeri*
Ehrlichia chaffeensis		

Table S8. *Relative abundance (> 0.01%) of pathogens.*

Phylum	Class	Order	Family	Genus	Species	Pipe_material	Cl	No Cl
Actinomycetota	Actinomycetia	Mycobacteriales	Mycobacteriaceae	Mycobacterium	Mycobacterium chelonae	Blank	0.01394653	5.70E-05
						PE	0.00121416	5.53E-05
						PPR	0.01429197	6.59E-05
						PVC	0.00092383	2.70E-05
Bacillota	Bacilli	Staphylococcales	Staphylococcaceae	Staphylococcus	Staphylococcus epidermidis	Blank	0.00335844	6.17E-05
						PE	0.00119983	6.80E-05
						PPR	0.00323858	5.21E-05
						PVC	0.00167076	9.34E-05
Pseudomonadota	Alphaproteobacteria	Caulobacterales	Caulobacteraceae	Brevundimonas	Brevundimonas vesicularis	Blank	0.00480108	7.32E-05
						PE	0.00573868	0.00276779
						PPR	0.26611027	0.00152549
						PVC	0.00320725	0.00466756
		Sphingomonadales	Sphingomonadaceae	Sphingomonas	Sphingomonas paucimobilis	Blank	0.02412731	0.03888005
						PE	0.10177341	0.05272059
						PPR	0.13673077	0.05411032
						PVC	0.1491174	0.14230624
	Gammaproteobacteria	Cardiobacteriales	Cardiobacteriaceae	Cardiobacterium	Cardiobacterium hominis	Blank	7.62E-05	0.0002456
						PE	1.68E-05	0.00014378
						PPR	0.01089272	1.45E-05
						PVC	3.96E-05	7.83E-05
		Enterobacterales_A	Aeromonadaceae	Aeromonas	Aeromonas veronii	Blank	0.00010009	2.62E-05
						PE	2.39E-05	2.01E-05
						PPR	9.57E-05	5.94E-05

					PVC	5.62E-06	2.22E-05
	Pseudomonadales	Moraxellaceae	Acinetobacter	Acinetobacter baumannii	Blank	0.00085306	2.27E-05
					PE	0.00367229	0.05083447
					PPR	0.00042282	0.07036491
					PVC	0.00949179	0.10618665
				Acinetobacter haemolyticus	Blank	0.00069092	2.83E-05
					PE	0.00561697	0.10163792
					PPR	0.00020528	0.14965743
					PVC	0.00687741	0.14576597
				Acinetobacter johnsonii	Blank	0.042475	0.00021984
					PE	0.08515248	0.57743366
					PPR	0.02278435	0.26063511
					PVC	0.25291879	5.51963046
				Acinetobacter junii	Blank	0.02290946	0.00165891
					PE	0.28603344	5.77784922
					PPR	0.00412822	8.69856414
					PVC	0.32655064	6.63588372
				Acinetobacter lwoffii	Blank	0.00082551	2.29E-05
					PE	0.00281201	0.03918148
					PPR	0.00034463	0.05387705
					PVC	0.00464231	0.08270399
				Acinetobacter radioresistens	Blank	0.00039755	/
					PE	0.00076537	0.00369224
					PPR	0.00024254	0.0012606
					PVC	0.00245302	0.02793055

	Xanthomonadales	Xanthomonadaceae	Stenotrophomonas	Stenotrophomonas maltophilia	Blank	0.01913942	0.00082586
					PE	0.01001487	0.00149957
					PPR	0.01925381	0.00331776
					PVC	0.00693537	0.00825839

Table S9. DOM molecules in Co-occurrence network.
Cl

Cl				No Cl			
Module	Formula	Element	Category	Module	Formula	Element	Category
0	C10H12O5	CHO	Lignin	0	C11H10O8	CHO	Tannins
0	C10H12O6	CHO	Lignin	0	C11H12O7Cl2	CHOCl	Lignin
0	C11H14O8	CHO	Tannins	0	C11H12O8	CHO	Tannins
0	C11H16O5	CHO	Lignin	0	C12H12O8	CHO	Lignin
0	C12H12O7	CHO	Lignin	0	C12H14O8	CHO	Lignin
0	C12H18O5	CHO	Lignin	0	C12H15O8N1	CHON	Lignin
0	C12H23O7N4P1	CHONP	Aminosugars	0	C12H16O5S1	CHOS	Lignin
0	C13H12O7	CHO	Lignin	0	C12H16O9S1	CHOS	Tannins
0	C13H14O10	CHO	Tannins	0	C12H17O7P1	CHOP	Lignin
0	C13H14O6	CHO	Lignin	0	C13H14O7	CHO	Lignin
0	C13H14O8	CHO	Lignin	0	C13H16O7	CHO	Lignin
0	C13H15O10P1	CHOP	Tannins	0	C13H20O8S1	CHOS	Others
0	C13H18O4	CHO	Lignin	0	C14H18O7	CHO	Lignin
0	C14H14O11	CHO	Tannins	0	C14H18O8	CHO	Lignin
0	C14H17O9P1	CHOP	Lignin	0	C15H10O10	CHO	ConAroStru
0	C14H18O5	CHO	Lignin	0	C15H16O10	CHO	Lignin
0	C14H18O7	CHO	Lignin	0	C15H16O7	CHO	Lignin
0	C14H18O9	CHO	Lignin	0	C15H17O9N1	CHON	Lignin
0	C15H16O10	CHO	Lignin	0	C15H18O8	CHO	Lignin
0	C15H17O10N1	CHON	Lignin	0	C15H19O10N1	CHON	Lignin
0	C15H17O7N1	CHON	Lignin	0	C15H20O9S1	CHOS	Lignin
0	C15H18O11	CHO	Tannins	0	C15H24O5S1	CHOS	N-FreeSatCom
0	C15H19O8N1	CHON	Lignin	0	C15H25O4P1	CHOP	N-FreeSatCom
0	C15H20O9	CHO	Lignin	0	C15H28O9S1	CHOS	Others
0	C15H21O10P1	CHOP	Lignin	0	C16H16O10	CHO	Lignin

0	C15H22O10	CHO	Lignin	0	C16H17O10N1	CHON	Lignin
0	C15H22O8	CHO	Lignin	0	C16H17O9N1	CHON	Lignin
0	C15H24O10	CHO	Others	0	C16H18O6	CHO	Lignin
0	C16H14O10	CHO	Lignin	0	C16H18O7	CHO	Lignin
0	C16H15O8N1	CHON	Lignin	0	C16H18O9	CHO	Lignin
0	C16H18O8	CHO	Lignin	0	C16H21O10P1	CHOP	Lignin
0	C16H19O10N1	CHON	Lignin	0	C16H24O9S1	CHOS	Lignin
0	C16H19O8N1	CHON	Lignin	0	C17H16O11	CHO	Lignin
0	C16H20O11	CHO	Tannins	0	C17H18O11S1	CHOS	Lignin
0	C16H20O7	CHO	Lignin	0	C17H19O12P1	CHOP	Tannins
0	C16H20O8	CHO	Lignin	0	C17H21O9N1	CHON	Lignin
0	C16H21O10N1	CHON	Lignin	0	C18H18O11	CHO	Lignin
0	C16H21O7N1	CHON	Lignin	0	C18H19O12P1	CHOP	Lignin
0	C16H23O10P1	CHOP	Lignin	0	C18H23O12P1	CHOP	Lignin
0	C16H23O8N1	CHON	Lignin	0	C19H18O11	CHO	Lignin
0	C16H24O10	CHO	Lignin	0	C19H18O13	CHO	Tannins
0	C16H24O11	CHO	Tannins	0	C19H26O10S1	CHOS	Lignin
0	C16H24O8	CHO	Lignin	0	C20H18O15	CHO	Tannins
0	C17H14O8	CHO	Lignin	0	C30H38O18	CHO	Lignin
0	C17H16O11	CHO	Lignin	0	C31H42O12	CHO	Lignin
0	C17H18O10	CHO	Lignin	0	C32H38O15	CHO	Lignin
0	C17H18O9	CHO	Lignin	0	C32H40O16	CHO	Lignin
0	C17H19O11P1	CHOP	Lignin	0	C35H42O16	CHO	Lignin
0	C17H20O10	CHO	Lignin	0	C35H54O11S1	CHOS	N-FreeSatCom
0	C17H20O12	CHO	Tannins	0	C36H44O18	CHO	Lignin
0	C17H20O9	CHO	Lignin	0	C37H46O21	CHO	Lignin
0	C17H21O8N1	CHON	Lignin	0	C13H24O6S1	CHOS	N-FreeSatCom
0	C17H22O7	CHO	Lignin	0	C16H30O9S1	CHOS	Others

0	C17H22O9	CHO	Lignin	0	C20H19O13N1	CHON	Lignin
0	C17H23O10N1	CHON	Lignin	0	C24H32O12S1	CHOS	Lignin
0	C17H23O9P1	CHOP	Lignin	0	C25H28O9	CHO	Lignin
0	C17H24O6	CHO	Lignin	1	C14H17O9N1	CHON	Lignin
0	C17H25O10N1	CHON	Lignin	1	C14H20O8	CHO	Lignin
0	C17H26O10	CHO	Others	1	C15H19O7N1	CHON	Lignin
0	C17H26O9	CHO	Others	1	C15H20O9	CHO	Lignin
0	C18H16O11	CHO	Lignin	1	C16H17O8N1	CHON	Lignin
0	C18H16O7	CHO	Lignin	1	C16H19O8N1	CHON	Lignin
0	C18H18O10	CHO	Lignin	1	C16H20O10	CHO	Lignin
0	C18H18O11	CHO	Lignin	1	C17H12O10	CHO	Lignin
0	C18H18O12	CHO	Lignin	1	C17H17O9N1	CHON	Lignin
0	C18H20O10	CHO	Lignin	1	C17H19O10N1	CHON	Lignin
0	C18H20O6	CHO	Lignin	1	C17H20O10N2	CHON	Lignin
0	C18H21O11N1	CHON	Lignin	1	C18H20O12	CHO	Lignin
0	C18H21O11P1	CHOP	Lignin	1	C18H22O10N2	CHON	Lignin
0	C18H22O10	CHO	Lignin	1	C18H24O9S1	CHOS	Lignin
0	C18H22O11	CHO	Lignin	1	C18H30O3S1	CHOS	N-FreeSatCom
0	C18H23O10N1	CHON	Lignin	1	C18H34O2	CHO	N-FreeSatCom
0	C18H23O9N1	CHON	Lignin	1	C19H18O10	CHO	Lignin
0	C18H24O10	CHO	Lignin	1	C19H19O11N1	CHON	Lignin
0	C18H25O11N1	CHON	Lignin	1	C19H21O12N1	CHON	Lignin
0	C18H26O12	CHO	Lignin	1	C19H24O13S1	CHOS	Tannins
0	C18H26O6	CHO	Lignin	1	C19H28O11S1	CHOS	Lignin
0	C18H26O9	CHO	Lignin	1	C19H30O3S1	CHOS	N-FreeSatCom
0	C18H28O10	CHO	Others	1	C20H20O16	CHO	Tannins
0	C18H28O11	CHO	Others	1	C20H20O9	CHO	Lignin
0	C18H28O9	CHO	N-FreeSatCom	1	C20H28O11S1	CHOS	Lignin

0	C19H18O10	CHO	Lignin	1	C20H28O13	CHO	Lignin
0	C19H18O12	CHO	Lignin	1	C20H28O9S1	CHOS	Lignin
0	C19H18O9	CHO	Lignin	1	C20H34O3S1	CHOS	N-FreeSatCom
0	C19H20O11	CHO	Lignin	1	C21H20O15	CHO	Tannins
0	C19H20O9	CHO	Lignin	1	C21H24O11S1	CHOS	Lignin
0	C19H22O10	CHO	Lignin	1	C21H24O12S1	CHOS	Lignin
0	C19H22O13	CHO	Tannins	1	C21H27O10N1	CHON	Lignin
0	C19H22O6	CHO	Lignin	1	C21H32O11S1	CHOS	Others
0	C19H24O10	CHO	Lignin	1	C22H30O12S1	CHOS	Lignin
0	C19H24O11	CHO	Lignin	1	C23H20O14	CHO	Lignin
0	C19H24O5	CHO	Lignin	1	C23H29O12N1	CHON	Lignin
0	C19H25O11N1	CHON	Lignin	1	C23H30O13S1	CHOS	Lignin
0	C19H26O10	CHO	Lignin	1	C23H31O12N1	CHON	Lignin
0	C19H27O10N1	CHON	Lignin	1	C23H32O11S1	CHOS	Lignin
0	C19H28O10	CHO	Lignin	1	C24H24O15	CHO	Lignin
0	C19H28O11	CHO	Lignin	1	C24H26O15	CHO	Lignin
0	C19H28O12	CHO	Lignin	1	C24H29O12N1	CHON	Lignin
0	C19H28O9	CHO	Lignin	1	C26H28O16	CHO	Lignin
0	C19H29O9P1	CHOP	N-FreeSatCom	1	C26H28O17	CHO	Lignin
0	C19H32O7	CHO	N-FreeSatCom	1	C27H34O15	CHO	Lignin
0	C20H20O10	CHO	Lignin	1	C27H34O16	CHO	Lignin
0	C20H20O11	CHO	Lignin	1	C27H36O14	CHO	Lignin
0	C20H20O12	CHO	Lignin	1	C28H36O14	CHO	Lignin
0	C20H22O10	CHO	Lignin	1	C29H30O16	CHO	Lignin
0	C20H22O11	CHO	Lignin	1	C29H34O14	CHO	Lignin
0	C20H22O13	CHO	Lignin	1	C29H36O14	CHO	Lignin
0	C20H22O8	CHO	Lignin	1	C29H36O15	CHO	Lignin
0	C20H24O10	CHO	Lignin	1	C29H36O17	CHO	Lignin

0	C20H24O13	CHO	Lignin	1	C29H38O15	CHO	Lignin
0	C20H24O7	CHO	Lignin	1	C29H44O10S1	CHOS	N-FreeSatCom
0	C20H24O9	CHO	Lignin	1	C30H34O16	CHO	Lignin
0	C20H26O10	CHO	Lignin	1	C30H40O15	CHO	Lignin
0	C20H26O11	CHO	Lignin	1	C31H38O12	CHO	Lignin
0	C20H26O9	CHO	Lignin	1	C31H38O18	CHO	Lignin
0	C20H27O9N1	CHON	Lignin	1	C31H44O16	CHO	Lignin
0	C20H28O10	CHO	Lignin	1	C32H38O14	CHO	Lignin
0	C20H28O11	CHO	Lignin	1	C32H42O16	CHO	Lignin
0	C20H28O12	CHO	Lignin	1	C32H44O14	CHO	Lignin
0	C20H30O11	CHO	Lignin	1	C32H44O16	CHO	Lignin
0	C20H30O9	CHO	Lignin	1	C32H50O10S1	CHOS	N-FreeSatCom
0	C21H22O13	CHO	Lignin	1	C33H42O15	CHO	Lignin
0	C21H22O8	CHO	Lignin	1	C34H46O15	CHO	Lignin
0	C21H24O10	CHO	Lignin	1	C35H52O12S1	CHOS	Lignin
0	C21H24O11	CHO	Lignin	1	C36H40O21	CHO	Lignin
0	C21H24O13	CHO	Lignin	1	C36H42O21	CHO	Lignin
0	C21H24O7	CHO	Lignin	1	C36H44O17	CHO	Lignin
0	C21H26O10	CHO	Lignin	1	C37H46O17	CHO	Lignin
0	C21H26O11	CHO	Lignin	1	C37H48O19	CHO	Lignin
0	C21H26O13	CHO	Lignin	2	C13H16O6S1	CHOS	Lignin
0	C21H26O9	CHO	Lignin	2	C17H18O8	CHO	Lignin
0	C21H28O10	CHO	Lignin	2	C20H26O5	CHO	Lignin
0	C21H28O11	CHO	Lignin	2	C20H32O11	CHO	Others
0	C21H28O7	CHO	Lignin	2	C21H18O10	CHO	Lignin
0	C21H28O9	CHO	Lignin	2	C21H26O10S1	CHOS	Lignin
0	C21H30O11	CHO	Lignin	2	C23H22O16	CHO	Tannins
0	C21H30O8	CHO	Lignin	2	C32H38O20	CHO	Lignin

0	C21H30O9	CHO	Lignin	2	C37H44O21	CHO	Lignin
0	C21H32O10	CHO	N-FreeSatCom	3	C13H19O8N1	CHON	Lignin
0	C22H22O12	CHO	Lignin	3	C14H10O10	CHO	Tannins
0	C22H24O11	CHO	Lignin	3	C14H19O6N1	CHON	Lignin
0	C22H24O13	CHO	Lignin	3	C16H15O11N1	CHON	Tannins
0	C22H26O10	CHO	Lignin	3	C17H20O9S1	CHOS	Lignin
0	C22H26O12	CHO	Lignin	3	C17H34O2	CHO	N-FreeSatCom
0	C22H26O13	CHO	Lignin	3	C18H20O14	CHO	Tannins
0	C22H26O9	CHO	Lignin	3	C19H14O11	CHO	Lignin
0	C22H28O11	CHO	Lignin	3	C19H20O12	CHO	Lignin
0	C22H28O13	CHO	Lignin	3	C19H20O13	CHO	Tannins
0	C22H30O10	CHO	Lignin	3	C20H18O12	CHO	Lignin
0	C22H30O8	CHO	Lignin	3	C21H23O11N1	CHON	Lignin
0	C22H32O11	CHO	Lignin	3	C22H25O11N1	CHON	Lignin
0	C22H32O8	CHO	Lignin	3	C22H34O9S1	CHOS	N-FreeSatCom
0	C22H32O9	CHO	Lignin	3	C23H25O14N1	CHON	Lignin
0	C23H24O13	CHO	Lignin	3	C23H27O12N1	CHON	Lignin
0	C23H26O11	CHO	Lignin	3	C23H30O12S1	CHOS	Lignin
0	C23H26O12	CHO	Lignin	3	C27H32O15	CHO	Lignin
0	C23H28O12	CHO	Lignin	3	C27H36O15	CHO	Lignin
0	C23H30O11	CHO	Lignin	3	C28H38O15	CHO	Lignin
0	C23H30O12	CHO	Lignin	3	C29H32O18	CHO	Lignin
0	C23H30O13	CHO	Lignin	3	C29H40O14	CHO	Lignin
0	C23H32O10	CHO	Lignin	3	C30H38O19	CHO	Lignin
0	C23H32O9	CHO	Lignin	3	C31H38O16	CHO	Lignin
0	C24H26O12	CHO	Lignin	3	C32H40O19	CHO	Lignin
0	C24H28O12	CHO	Lignin	3	C33H42O19	CHO	Lignin
0	C24H30O12	CHO	Lignin	3	C34H46O14	CHO	Lignin

0	C24H32O11	CHO	Lignin	3	C34H46O19	CHO	Lignin
0	C24H32O9	CHO	Lignin	3	C35H50O15	CHO	Lignin
0	C24H36O9	CHO	Lignin	3	C40H50O19	CHO	Lignin
0	C25H28O12	CHO	Lignin	3	C40H50O20	CHO	Lignin
0	C25H30O13	CHO	Lignin	3	C41H52O20	CHO	Lignin
0	C25H32O14	CHO	Lignin	3	C9H17O8N4P1	CHONP	Carbohydrates
0	C25H34O10	CHO	Lignin				
0	C25H34O11	CHO	Lignin				
0	C25H36O12	CHO	Lignin				
0	C26H34O12	CHO	Lignin				
0	C26H34O13	CHO	Lignin				
0	C26H36O12	CHO	Lignin				
0	C26H38O11	CHO	Lignin				
0	C27H34O10	CHO	Lignin				
0	C27H36O11	CHO	Lignin				
0	C27H38O12	CHO	Lignin				
0	C28H40O11	CHO	Lignin				
0	C28H43O4P1	CHOP	N-FreeSatCom				
0	C35H50O13S1	CHOS	Lignin				
0	C36H54O13S1	CHOS	Lignin				
0	C9H12O6	CHO	Lignin				
0	C12H26O3S1	CHOS	N-FreeSatCom				
0	C8H18O3S1	CHOS	Others				
0	C8H18O3S2	CHOS	Others				
1	C14H17O10P1	CHOP	Tannins				
1	C18H21O10P1	CHOP	Lignin				
1	C20H38O9	CHO	N-FreeSatCom				
1	C31H50O8S1	CHOS	N-FreeSatCom				

1	C32H52O8S1	CHOS	N-FreeSatCom
2	C27H42O9S1	CHOS	N-FreeSatCom
2	C34H52O12S1	CHOS	N-FreeSatCom
2	C35H54O12S1	CHOS	N-FreeSatCom
2	C38H48O7S2	CHOS	Lignin

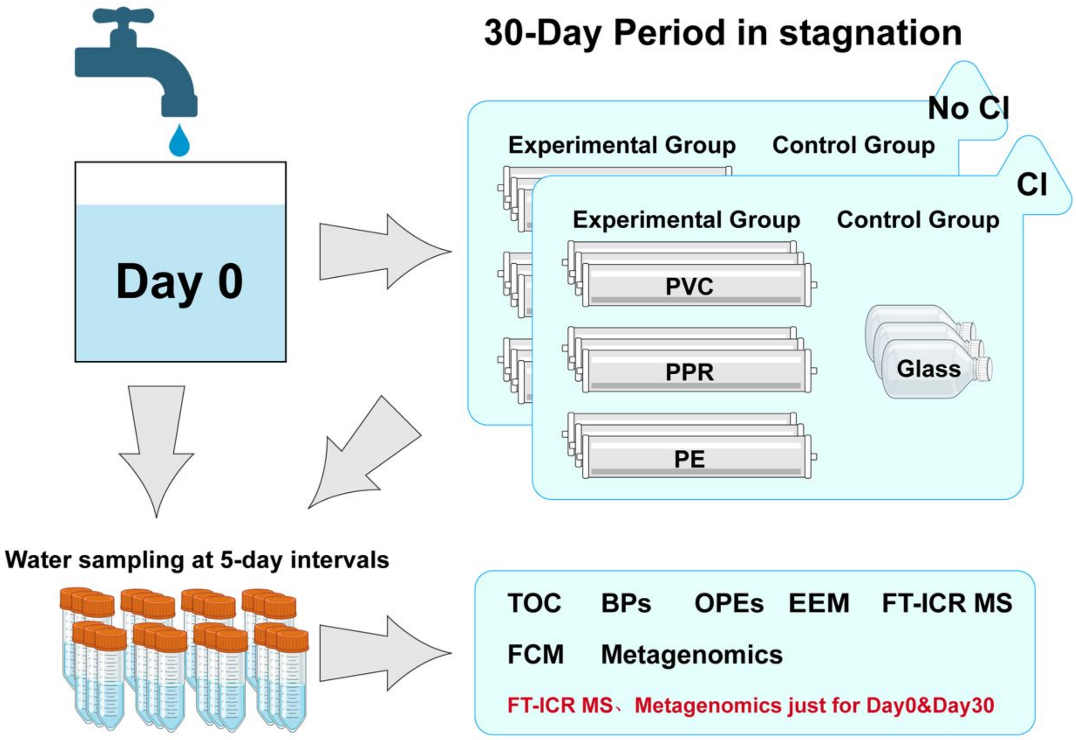

Figure S1. Experimental Schematic for Exploring Plastic Leachate Migration under Chlorinated and Non-Chlorinated Conditions

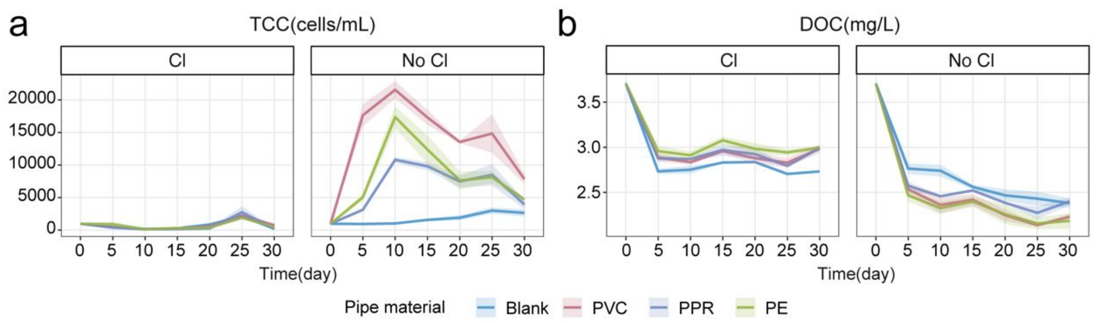

Figure S2. *Changes of biomass quantified by (a) total cell count (TCC) and (b) dissolved organic carbon concentrations (DOC). Lines with ribbons indicate means ± standard error (SE) (n = 3).*

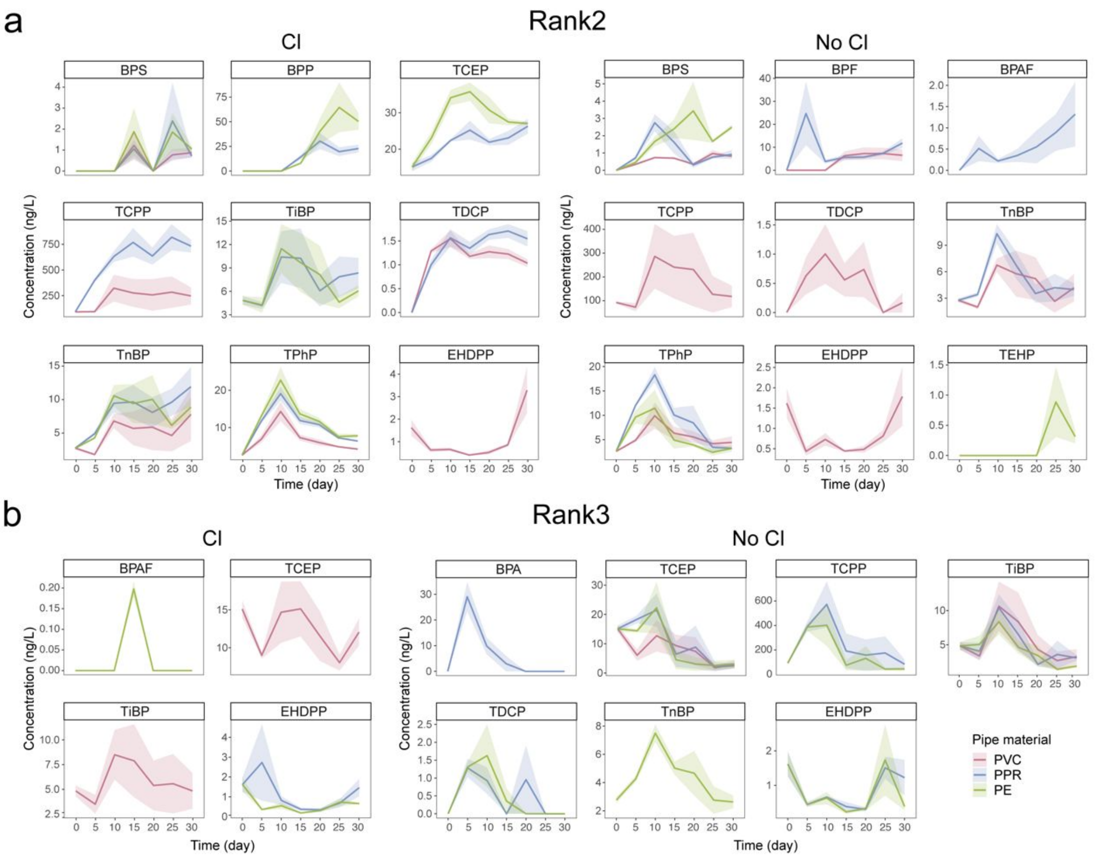

Figure S3. *Changes of organic additives concentration of (a)Rank2 and (b)Rank3. Lines with ribbons indicate means ± standard error (SE) (n = 3).*

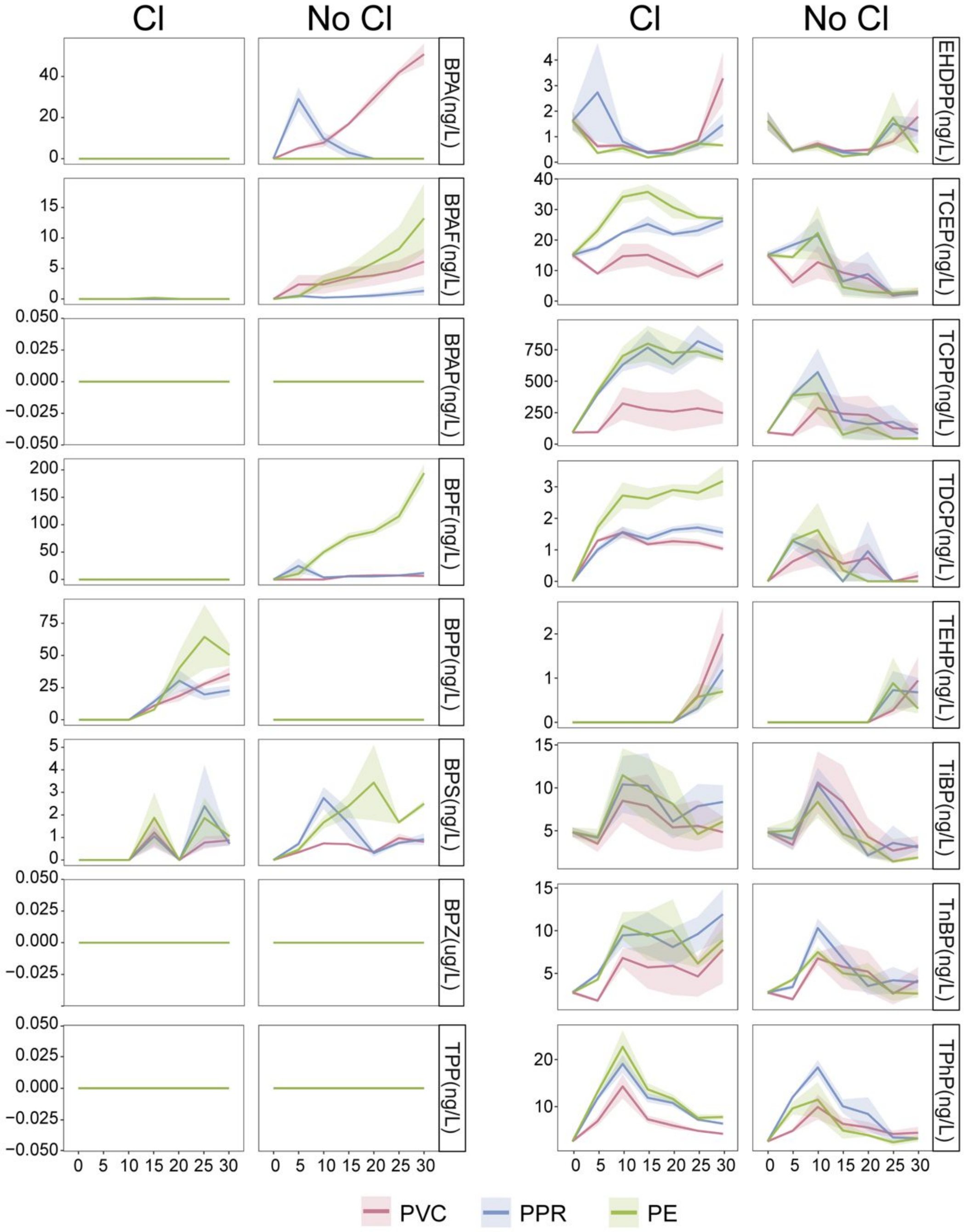

Figure S4. *Comparison of plastic additives concentration changes under chlorinated and non-chlorinated conditions. Lines with ribbons indicate means ± standard error (SE) (n = 3).*

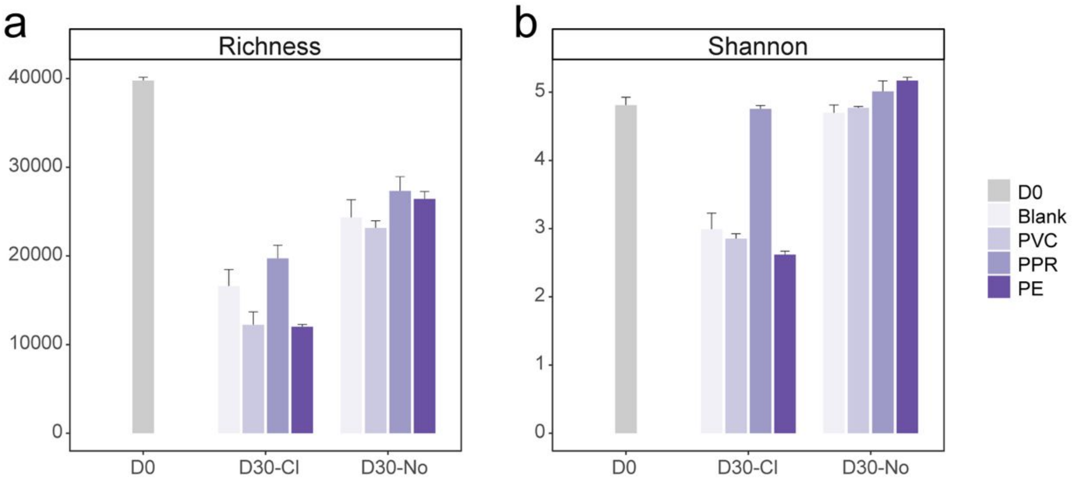

Figure S5. *Bar plot of the (a) richness and (b) alpha diversity (Shannon) of bacterial community. Error bars represent means ± standard error (SE) (n = 3).*

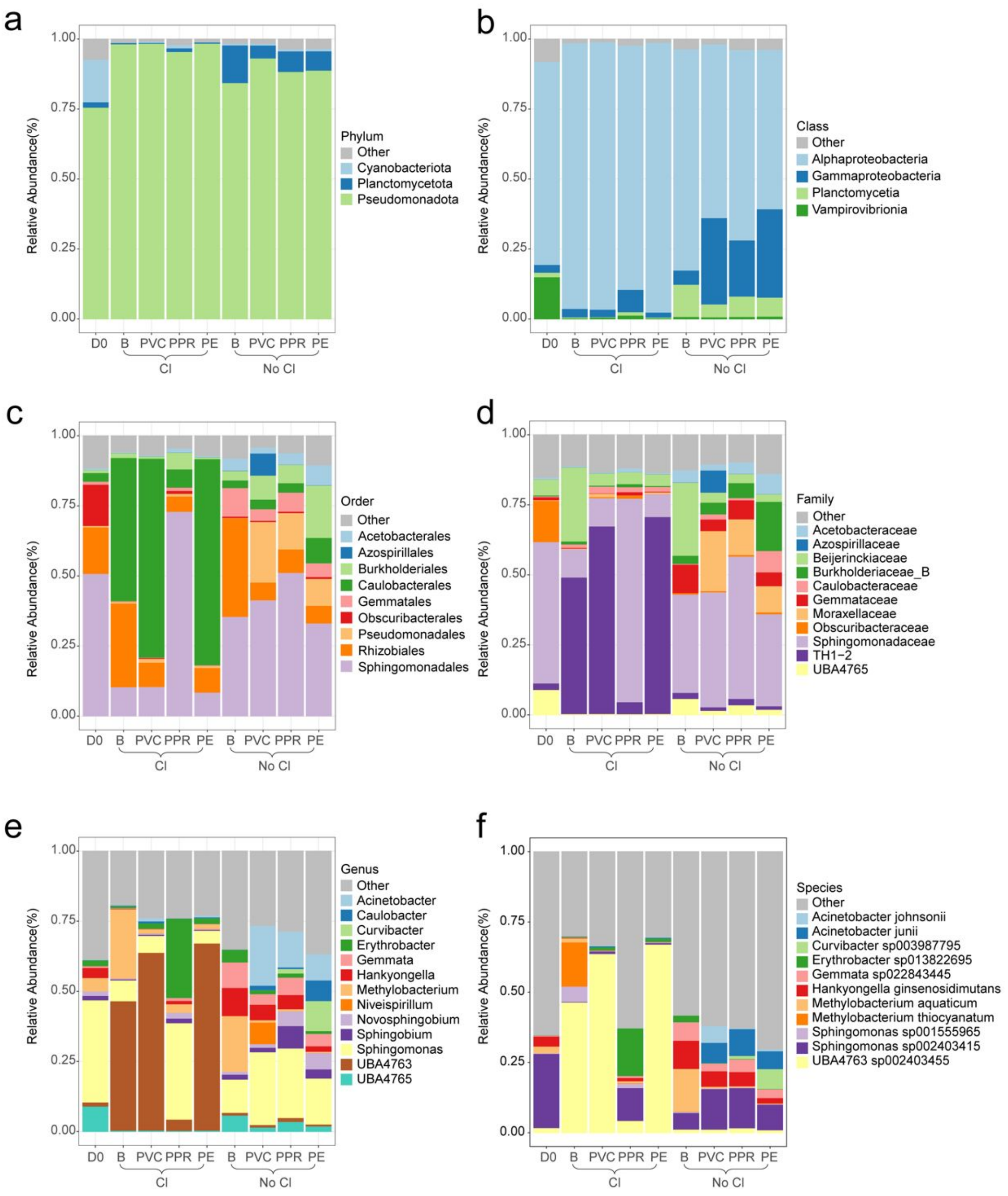

Figure S6. *Changes in bacterial community composition at (a)Phylum, (b)Class, (c)Order, (d)Family, (e)Genus and (f)Species level during experiment. Taxa with relative abundances below 0.05% are shown as Other.*

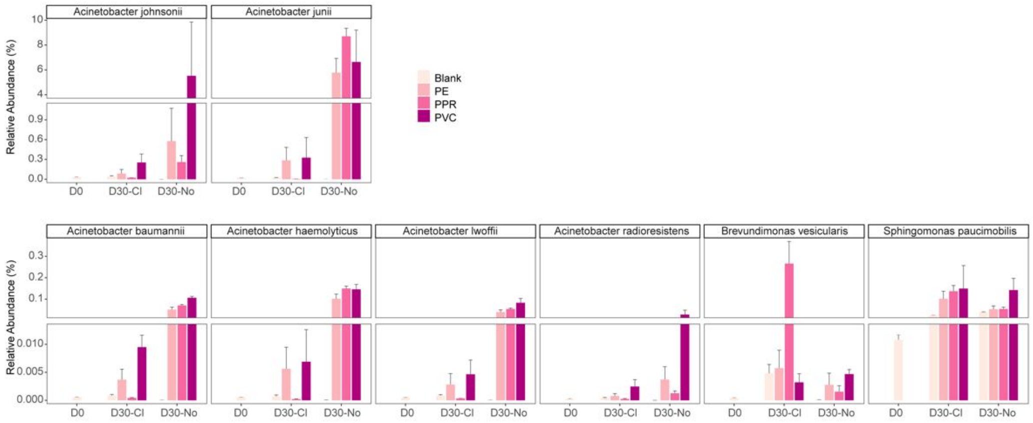

Figure S7. *Bar plot of the relative abundance of plastic enriched pathogens. Error bars represent means ± standard error (SE) (n = 3).*

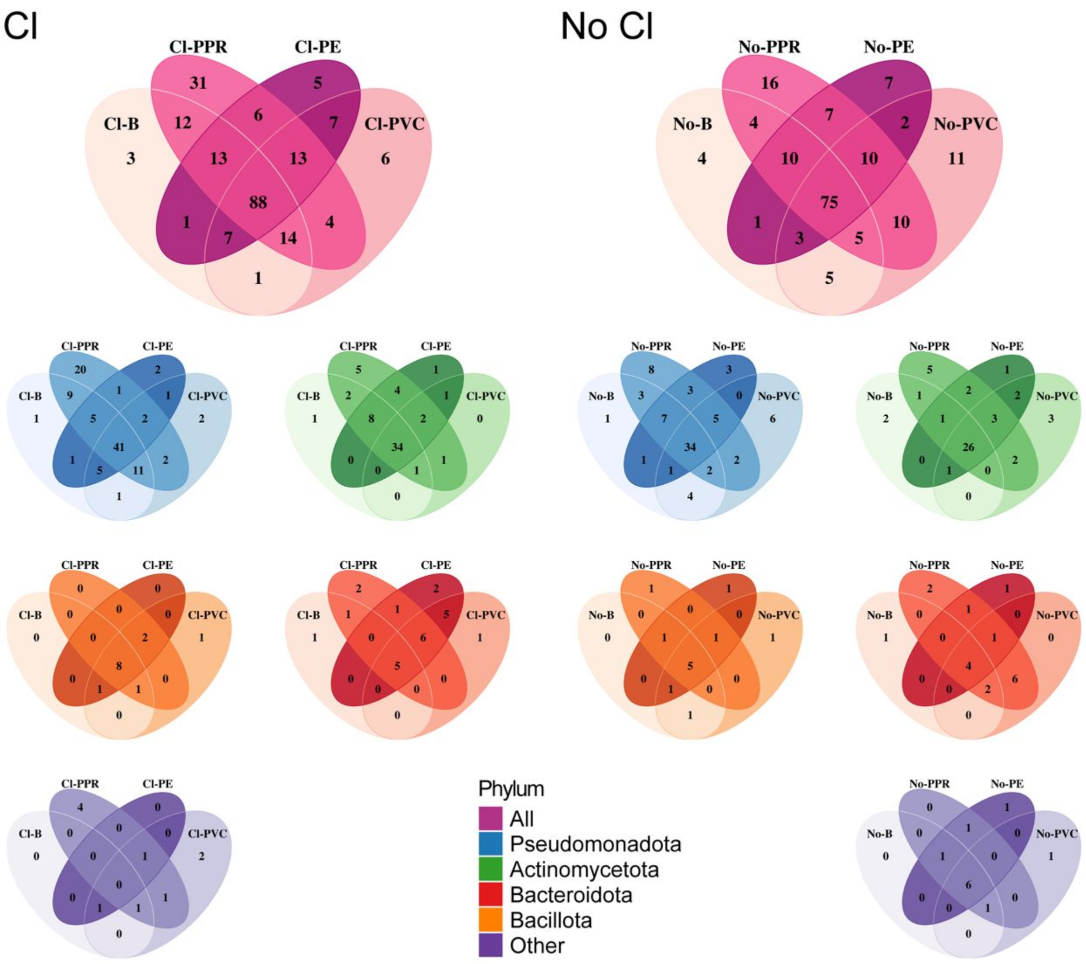

Figure S8. *Venn diagram of the number of shared and unique pathogens among different pipe materials.*

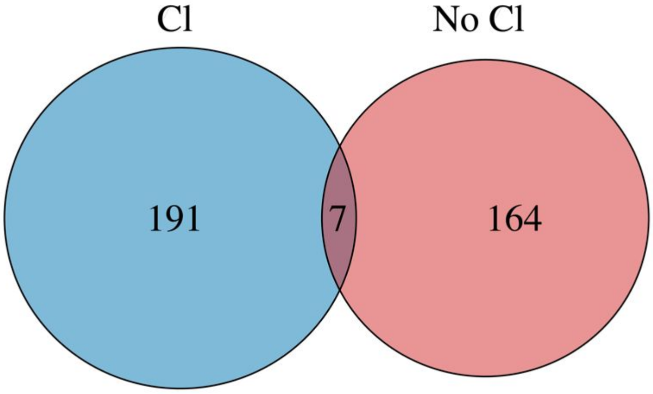

Figure S9. *Venn diagram of the number of chlorinated and non-chlorinated DOM molecules in Co-occurrence network.*

Reference

1 Fu, Q. L., Fujii, M. & Kwon, E. Development and Application of a High-Precision Algorithm for Nontarget Identification of Organohalogens Based on Ultrahigh-Resolution Mass Spectrometry. *Anal Chem* **92**, 13989-13996 (2020). https://doi.org:10.1021/acs.analchem.0c02899
